# Air-stable n-type dopant for organic semiconductors via a single-photon catalytic process

**DOI:** 10.1126/sciadv.adu8215

**Published:** 2025-06-06

**Authors:** Liang Yan, Xinzheng Yang, Mengqi Yang, Justin Neu, Somayeh Kashani, Rajiv Giridharagopal, Yusuf Olanrewaju, Franky So, David Ginger, Harald Ade, Xiaosong Li, Wei You

**Affiliations:** ^1^ Department of Chemistry, University of North Carolina at Chapel Hill, Chapel Hill, NC 27599, USA.; ^2^ Department of Chemistry, University of Washington, Seattle, WA 98195, USA.; ^3^ Department of Electrical and Computer Engineering and ORaCEL, North Carolina State University, Raleigh, NC 27695, USA.; ^4^ Department of Materials Science and Engineering, North Carolina State University, Raleigh, NC 27695, USA.; ^5^ Department of Physics and ORaCEL, North Carolina State University, Raleigh, NC 27695, USA.

## Abstract

Controlled doping of conjugated polymer-based semiconductors is crucial for optoelectronic applications. While p-type doping of conjugated polymers can be readily achieved with a variety of oxidants, n-type doping is more challenging, usually requiring highly reactive reducing agents. Here, we demonstrate that an air-stable photoredox catalyst (acridinium salt), together with a mild and air-stable reducing agent (amine), can effectively dope common n-type conjugated polymers under light at room temperature, yielding conductivity values on par with the highest obtained via other means. We elucidate the mechanism and show that this photoredox n-doping occurs via a one-photon–one-electron transfer process that is catalytic in nature. This simple and facile n-doping approach opens more avenues for doping organic semiconductors with the potential to revolutionize device design and substantially enhance doping efficiency.

## Teaser

Photoredox catalyst with a mild reductant can n-type dope conjugated polymers via a catalytic one-photon–one-electron process.

## INTRODUCTION

Molecular doping in organic semiconductors (e.g., conjugated polymers) is the process of generating free positive (or negative) charge carriers in organic semiconductors by using molecular oxidizing (or reducing) agents (*1*, *2*). Molecular doping plays an instrumental role in key applications of organic electronics, including commercial organic light-emitting diodes (*3*), photovoltaics (*4*–*6*), and thermoelectrics (*7*–*9*). Molecular doping in conjugated polymers is usually achieved by strong oxidants [e.g., F4TCNQ and Mo(tfd)_3_] or strong reductants (e.g., lithium metal) to maximize the achievable conductivity in doped conjugated polymers. Generally, p-doping can be readily achieved with a variety of oxidants (including molecular oxygen for conjugated polymers having low ionization potentials, IP); by contrast, only a few air-stable n-dopants have been reported (*10*–*12*) because strong reducing agents (e.g., Li) are fundamentally unstable toward air (e.g., oxygen). The most prominent air-stable n-dopants are benzoimidazole-based hybrid molecules (e.g., NDBMI-H) that undergo a C-H cleavage reaction to generate the active doping species in situ (*11*, *13*).

Photoredox catalysis, where an excited state of a metal complex or organic dye molecule—upon photoexcitation—shuttles electrons to enable highly challenging or energetically forbidden organic reactions in a light-driven catalytic cycle, has gained substantial momentum in the past 15 years (*14*–*17*). Photoredox catalysis uses photoredox catalysts that are stable in the ground state and usually weak redox species; however, when excited by light (for example, with simple household lightbulbs), these benign photoredox catalysts can turn into a potent oxidant or reductant that can accomplish the targeted organic transformation in a catalytic manner (*18*). The unique features of photoredox catalysis have been very recently extended to the doping of conjugated polymers; Jin *et al.* (*19*) reported the successful doping of a variety of p-type conjugated polymers, particularly facilitated by air-stable acridinium-based photoredox catalysts. Because the acridinium-based photoredox catalysts can undergo a two-photon–two-electron process to form a very strong reductant (as strong as lithium) and only require a catalytic amount in the acridinium-catalyzed organic reactions (*18*), Jin *et al.* proposed a similar two-photon–two-electron mechanism to account for the observed doping of conjugated polymers and assumed a plausible catalytic process; yet, this mechanism has not been experimentally validated.

Here, we show that a commercially available and air-stable acridinium salt [9-mesityl-3,6-di-*tert*-butyl-10-phenylacridinium tetrafluoroborate (Mes-Acr^+^BF_4_^−^)], when paired with an air-stable mild reductant [e.g., tertiary amine, *N*,*N*-diisopropylethylamine (DIPEA)] as the dopant, can efficiently dope several common n-type conjugated polymers [e.g., poly{[*N*,*N*′-bis(2-octyldodecyl)naphthalene-1,4,5,8-bis(dicarboximide)-2,6-diyl]-alt-5,5′-(2,2′-bithiophene)} (N2200) and poly(benzimidazobenzophenanthroline) (BBL)]. We show that this photoredox catalyst–enabled n-type doping is accomplished via a one-photon–one-electron process. Specifically, upon ultraviolet (UV)/blue-light excitation, the excited Mes-Acr^+*^ species undergoes one-electron transfer from the amine (dopant) and converts into Mes-Acr^•^; the latter has a sufficiently high reducing potential to enable electron transfer to the conjugated polymer via a tunneling-based process, and returns to the ground state (Mes-Acr^+^) with the conjugated polymer being n-doped (e.g., N2200^•−^). Further, we validate the catalytic nature of photoredox catalyst–based doping, which is controlled by diffusion during the dip-doping process.

## RESULTS

### Photoredox catalyst–based n-doping of N2200

**Fig. 1.**
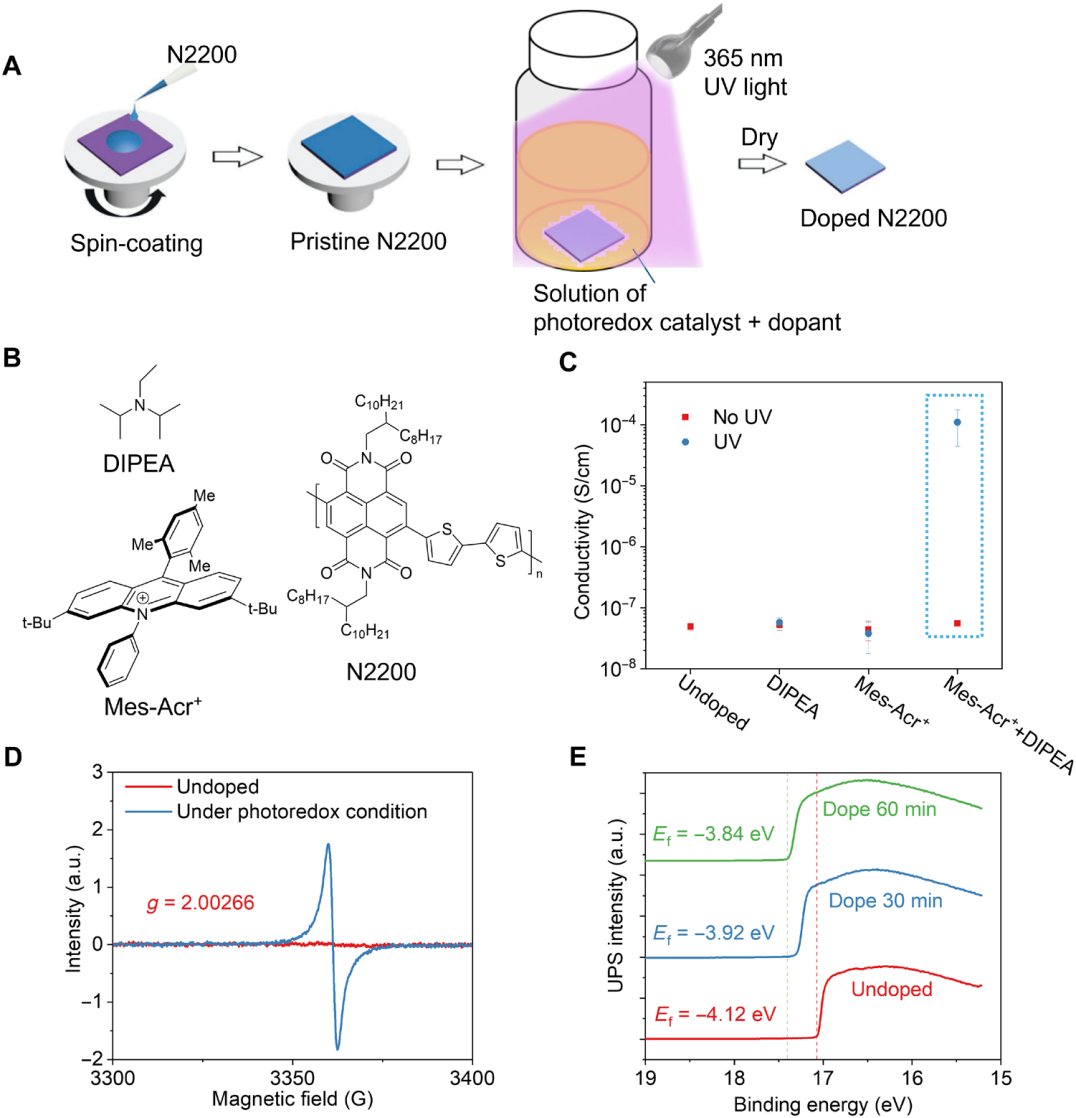
Photoredox catalyst–based doping for N2200. (**A**) Illustration of the dip doping method used for the photoredox catalyst–based doping (Mes-Acr^+^: 1 mg/ml, DIPEA: 1 μl/ml, UV illumination for a set amount of time). (**B**) Chemical structure of DIPEA, Mes-Acr^+^, and N2200. (**C**) The measured conductivity of N2200 films under different conditions with 30 min of UV light illumination. (**D**) EPR spectrum of N2200 films before and after the dip doping for 30 min under the photoredox condition. (**E**) Fermi level (*E*_f_) of dip doped N2200 film under the photoredox condition.

We chose N2200 as a model n-type conjugated polymer, as N2200 and its derivatives are perhaps the most studied n-type conjugated polymers for n-doping (*20*, *21*). When doped by NDMBI-H, N2200 can achieve conductivities on the order of 10^−3^ S/cm (*22*). We chose the photoredox catalyst, Mes-Acr^+^BF_4_^−^ (hereafter, Mes-Acr^+^, or Acr^+^) (*18*), together with the amine DIPEA, to facilitate n-type doping of N2200 under UV light. Given that photoredox catalysis for organic reactions are carried out in solution, yet doping is typically conducted with thin films of conjugated polymers, we applied a “photo-dip-doping” process in our doping experiments. As illustrated in Fig. 1A, we first dissolve N2200 in chloroform and then spin-coated a thin film (~100 nm thick) onto a glass substrate. Subsequently, we submerge the N2200 thin film in an acetonitrile solution containing both Mes-Acr^+^ and DIPEA under 365 nm of UV light. After a set time (e.g., 30 min), we remove the N2200 film from the dopant solution and dry the doped N2200 film using nitrogen gas. As shown in Fig. 1C, we observe a substantial increase in conductivity when both Mes-Acr^+^ and DIPEA are present in the solution under UV illumination. When only one component (either Mes-Acr^+^ or DIPEA) is present, or when there is no UV illumination, we only observe negligible changes of the conductivity of the N2200 films. Thus, all three components (Mes-Acr^+^, DIPEA, and UV) are required to achieve effective photoredox catalyst–based n-doping of N2200.

To understand the generation of polarons in the doping process, we conducted electron paramagnetic resonance (EPR) analysis on the N2200 films before and after dip doping under the photoredox condition. As expected, films subjected to dip doping for 30 min exhibit a strong EPR signal (Fig. 1D) whereas the pristine films before doping show no EPR signals. The *g* factor of the EPR signal of the dip-doped N2200, at 2.00266, is close to that of a free electron, consistent with the presence of free polarons in the dip-doped N2200 film. Furthermore, UV photoelectron spectroscopy (UPS) and Kelvin probe measurements (fig. S2) reveal a gradual shift of the Fermi level of the doped N2200 toward the vacuum level [or lowest unoccupied molecular orbital (LUMO) level of N2200] with increasing doping time when compared with the undoped reference (Fig. 1E), confirming that n-type doping indeed occurs in the N2200 films doped under the photoredox condition. When we add an ionic liquid, 1-butyl-1-methylpyrrolidinium bis(trifluoromethylsulfonyl)imide (BMP-TFSI), to the system, at a molar ratio of 10:1 to Mes-Acr^+^ to perform ion exchange (*23*), we improve the conductivity further to 10^−3^ S/cm (fig. S3A), close to the highest conductivity achieved for n-doped N2200 (*22*, *24*). We note that ionic liquids alone cannot dope N2200 in the absence of the three components (Mes-Acr^+^, DIPEA, and UV, fig. S3B) as ionic liquids only facilitate the ion exchange with the counterions (from the primary dopants to BMP^+^ in our case).

### Generality of photoredox-based n-doping

**Fig. 2.**
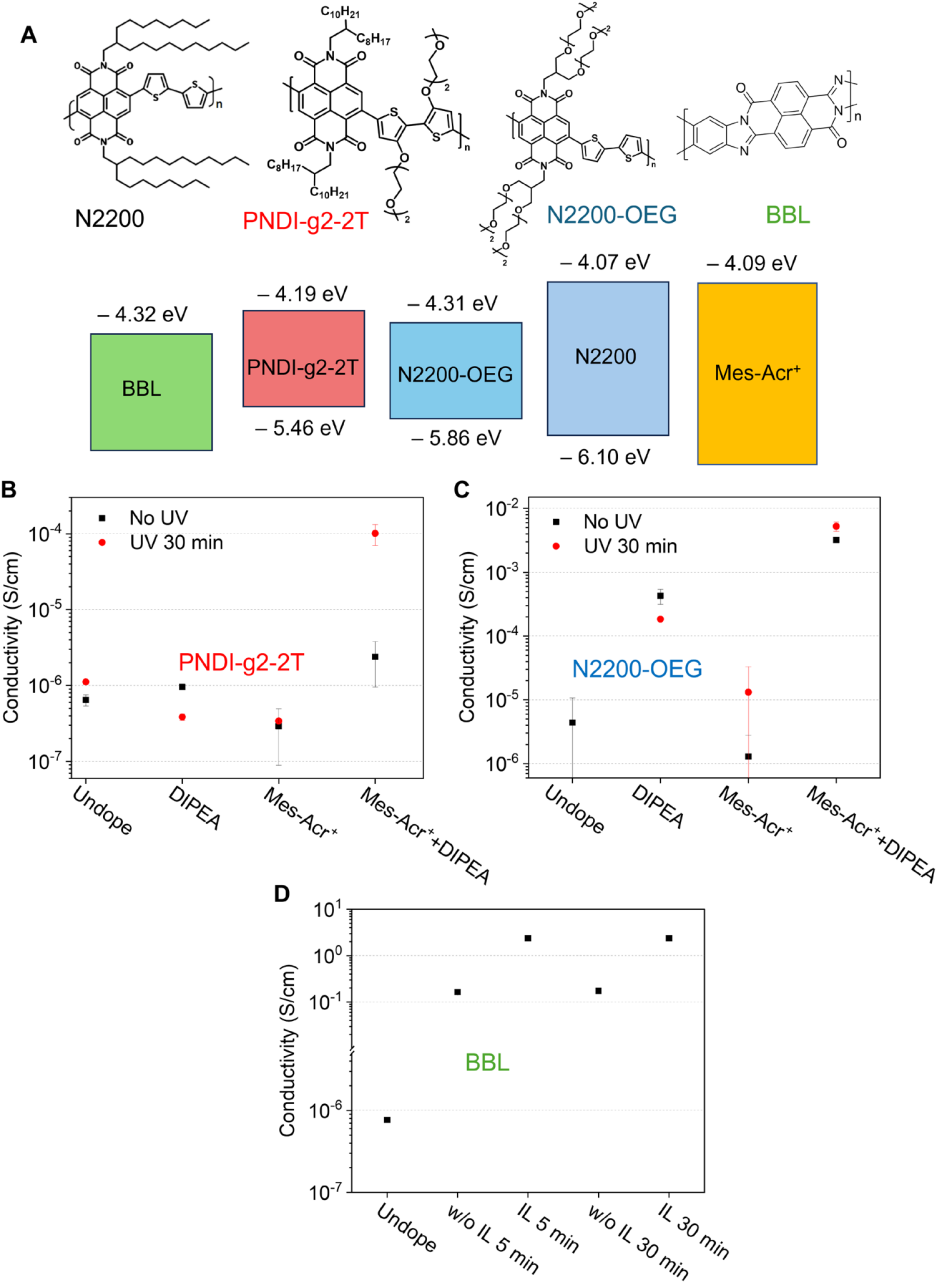
Photoredox catalyst–based n-doping for different polymers. (**A**) Chemical structure and energy levels of studied n-type polymers. (**B** to **D**) Dip doping (UV during dipping) results for PNDI-g2-2T (B), N2200-OEG (C), and BBL (D). In all cases, initial concentration was Mes-Acr^+^: 1 mg/ml, DIPEA: 1 μl/ml, Mes-Acr^+^:IL (BMP-TSFI) = 1:10 in the samples with IL.

To evaluate the generality of this photoredox catalyst–based n-doping process, we further study the impact of altering the side chains and the backbone of n-type conjugated polymers. Oligo(ethylene glycol) (OEG)–based side chains have been shown to improve the obtained conductivity of n-type conjugated polymers (*25*–*27*), ascribed to the better miscibility of the ionized dopant (usually also becoming the counterions). We thus synthesized two different versions of OEG-N2200 polymers, where OEG side chains are on the naphthalenediimide (NDI) unit (i.e., N2200-OEG) or on the bi-thiophene unit (PNDI-g2-2T). On the other hand, we also chose BBL, a classical n-type material with a ladder-like conjugated backbone that has shown a great potential for n-doped conjugated polymers in organic field-effect transistors (*28*) and organic electrochemical transistors (*29*). Figure 2A shows the chemical structures of those polymers and their LUMO/highest occupied molecular orbital energy levels [determined by cyclic voltammetry (CV), fig. S4].

The experimental results for PNDI-g2-2T closely resemble those of N2200: We only observe a substantial increase of conductivity when all three—Mes-Acr^+^, DIPEA, and UV illumination (Fig. 2B)—are present. In the case of N2200-OEG, DIPEA alone is already able to dope the conjugated polymer slightly (Fig. 2C); however, we obtain a substantial increase in conductivity (up to almost ~10^−2^ S/cm) under the photoredox condition (Mes-Acr^+^ and DIPEA under UV illumination). These results indicate that the photoredox catalyst substantially enhances the doping efficiency of DIPEA, a relatively weak electron donor.

Using BBL, on the other hand, we realize much higher conductivities (more than 0.1 cm/S, Fig. 2D) under the standard photoredox condition (Mes-Acr^+^, DIPEA, and UV). In addition, adding an ionic liquid (BMP-TFSI) further improves the conductivity by approximately one order of magnitude, reaching a value of 1 S/cm. Variations in dipping time, specifically 5 min versus 30 min, do not notably affect the conductivity of doped BBL, indicating that the diffusion process (to be discussed later) might be much faster in BBL than in N2200.

### Single-photon, single-electron process

As stated earlier, MacKenzie *et al.* (*18*) applied the photoredox condition (Mes-Acr^+^, DIPEA, and UV) to accomplish energetically demanding organic reactions (e.g., reducing chlorobenzene to benzene) and proposed a two-photon–based process to offer the required strong reducing power; this two-photon–based process was invoked by Jin *et al.* (*19*) in their photoredox catalyst–based doping process. In the two-photon–based process operated in our doping experiment, after the first photon to convert Mes-Acr^+^ into Mes-Acr^•^, this radical could be further excited by the second photon to a higher-energy twisted intramolecular charge-transfer (TICT) state, which could then facilitate the electron transfer to N2200. However, after we measured energy levels of all our materials by CV (fig. S4 and tables S1 and S2), we discover that the measured energy level (IP) of 4.09 eV for Mes-Acr^•^—generated by the first photon in the presence of DIPEA—is already sufficient to induce the electron transfer to N2200, which has an electron affinity (EA) of 4.07 eV. To differentiate these two different processes (two-photon versus one-photon), we conducted control experiments, and the results suggest that the single-photon and single-electron transfer pathway dominates the photoredox catalyst–based doping.

The reported TICT state has a short lifetime of approximately 100 ps (*18*), which should dissipate almost immediately upon termination of UV exposure, leaving only the radical state (Mes-Acr^•^) in solution. In our first control experiment, we mixed Mes-Acr^+^ with DIPEA in acetonitrile and the solution was illuminated by UV light for 1 hour. After the UV light was turned off, we then dipped the N2200 film into the “activated” solution for 30 min (this process is referred to as “UV before dipping”). Despite the absence of active UV illumination during doping, we observe a substantial increase in the conductivity of the doped N2200 film (fig. S5), similar to that we obtained above (i.e., “UV during dipping”). This observation suggests that the radical state (Mes-Acr^•^) alone is capable of doping N2200. We observe similar behavior in the case of n-doping BBL as well. When the solution containing both Mes-Acr^+^ and DIPEA was exposed to UV light before the dipping of BBL (i.e., UV before dipping), we measured similar conductivity (more than 0.1 cm/S, fig. S6) to that from the UV-during-dipping condition (Fig. 2D), further suggesting that TICT state does not contribute much to the doping of BBL under these conditions.

Because the TICT state can only be accessed by high-energy UV light while lower-energy blue light is only able to convert Mes-Acr^+^ into Mes-Acr^•^ (*18*), our second control experiment applied blue light (34 W Kessil H150-blue, fig. S7A) during the doping process of the N2200 film under the photoredox condition (UV during dipping). We observe similar increases in the conductivity of the doped N2200 film (fig. S7B) with both UV and blue illumination, again indicating that the TICT state is not essential for achieving n-type doping of conjugated polymers.

The proposed TICT state has a reducing power as strong as lithium (*18*) and should be able to dope organic semiconductors of low EAs, e.g., poly(9,9-dioctylfluorene-alt-benzothiadiazole) (F8BT, EA of 3.38 eV, table S2) and Indene-C60 bisadduct (ICBA, EA of 3.97 eV, table S2). However, in our third control experiment, we were unable to observe any noticeable doping of these organic semiconductors (fig. S8), which again indicates minimal contribution of the TICT state to the photoredox catalyst–based n-doping of conjugated polymers in this study.

**Fig. 3.**
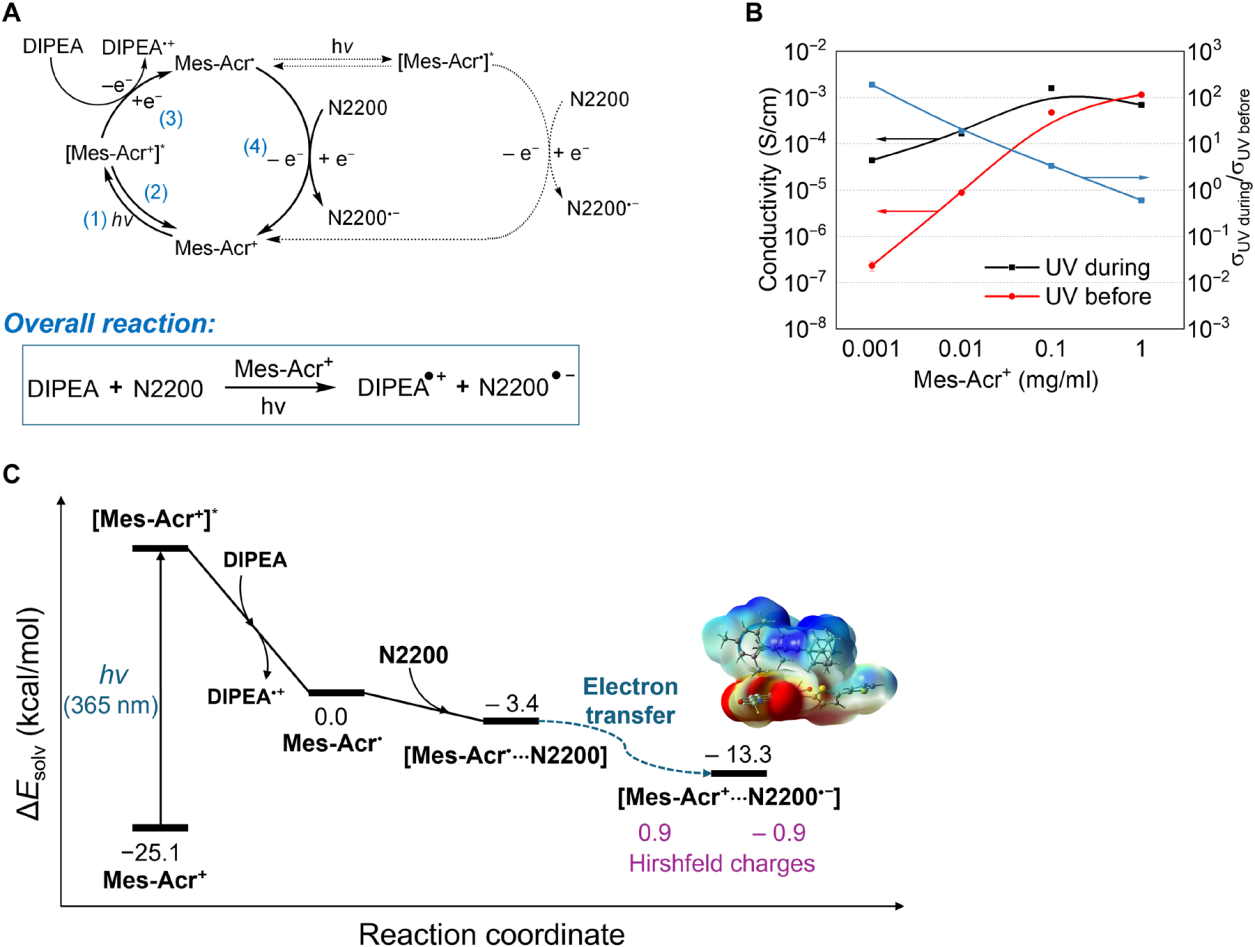
Mechanism of the photoredox catalyst–based n-type doping process. (**A**) Mechanism of n-type doping of N2200 under the photoredox condition; the dotted line represents the unlikely “two-photon–two-electron” process. (**B**) Comparing the difference in conductivity between UV before dipping and UV during dipping as varying the Mes-Acr^+^ concentration. DIPEA at 1 μl/ml and BMP-TFSI at 5.33 μl/ml were kept constant, except in the case of Mes-Acr^+^ at 1 mg/ml where more BMP-TFSI was used. The relative molar ratio of Mes-Acr^+^:DIPEA:IL changes from (i) 1:3.3:100, (ii) 0.1:3.3:10, (iii) 0.01:3.3:10, to (iv) 0.001:3.3:10, correspondingly. In (i), the quantity of Mes-Acr^+^ was ~100 times the number of repeat units of N2200 in its thin film; from (ii) to (iv), Mes-Acr^+^ was reduced to ~10×, 1×, and 0.1× the number of the repeat units of N2200 (all N2200 thin films were controlled to be ~100 nm and 1.5 ml of doping solution was used for i to iv). (**C**) Mechanistic reaction pathway for the doping process. Relative energy levels are depicted in reference to the noninteracting Mes-Acr^•^ and N2200 complex.

The accumulated experimental evidence reveals that the single-photon, single-electron process dominates the photoredox catalyst–based n-type doping; we thus infer the catalytic cycle illustrated in Fig. 3A (*14*). Specifically, Mes-Acr^+^ is first excited upon UV exposure to its excited state Mes-Acr^+*^, which then accepts an electron from DIPEA to form the radical Mes-Acr^•^ (and concurrently DIPEA^•+^). This highly reductive radical (with an energy level of 4.09 eV as estimated by CV, fig. S4) subsequently transfers an electron to N2200 to achieve proximal n-doping (i.e., N2200^•−^) and regenerates the Mes-Acr^+^ (ground state) for the next catalytic cycle. The entire process practically enables DIPEA doping N2200 with Mes-Acr^+^ as the catalyst, powered by UV light, according to this overall process

$$\begin{array}{c} \text{DIPEA}+N2200\;\overset{\text{Mes}‐{\text{Acr}}^{+}}{\underset{\text{hv}}{\to }}{\text{DIPEA}}^{•+}+N2200^{•-} \end{array}$$

### Catalytic nature

The one-photon–one-electron mechanism delineated in Fig. 3A implies a catalytic process that has not been validated for photoredox catalysis–based doping (*19*). To elucidate the mechanism, we compare UV exposure before dipping versus during dipping. Under the UV-before-dipping condition, the generated Mes-Acr^•^ should be equivalent to the amount of Mes-Acr^+^ for long enough exposure because only the left half of the catalytic cycle (steps 1 through 3 in Fig. 3A) can be accomplished under UV. A higher concentration of Mes-Acr^•^ should lead to a higher diffusion rate (to be discussed later) and thereby an increased doping level of N2200 for a fixed experimental time. By contrast, under the UV-during-dipping condition, a complete catalytic cycle should regenerate Mes-Acr^+^ and replenish Mes-Acr^•^ to continuously dope N2200 under UV exposure. Thus, the initial concentration of Mes-Acr^+^ should have less impact on the observed conductivity of doped N2200 (if the process is not diffusion limited). Shown in Fig. 3B, when we vary the amount of photoredox catalyst by 10^3^, we observe a difference in conductivity of more than 10^3^ under the UV-before-dipping condition but only a difference of 15 times in conductivity under the UV-during-dipping condition. While the ratio of conductivity under UV during dipping versus UV before dipping is only ~3 in the case of high concentration of Mes-Acr^+^ (0.1 mg/ml) and is close to 1 for even higher concentration (1 mg/ml), this ratio becomes ~200 under the lowest concentration (0.001 mg/ml), clearly indicating the catalytic nature of the photoredox catalyst–based n-doping of N2200.

### Electron transfer mechanism

We further carried out density functional theory (DFT) and real-time time-dependent DFT (RT-TDDFT)–based computational studies to shed more light on our proposed mechanism, with the calculated mechanistic reaction pathway for the dip doping process illustrated in Fig. 3C (see the Supplementary Materials for computation details). Our calculations indicate that the Mes-Acr^•^ radical and N2200 can form a neutral [Mes-Acr^•^···N2200] complex via dispersion interactions, as well as a slightly more stable [Mes-Acr^+^···N2200^•−^] complex. The latter is more stable due to coulombic attraction between Mes-Acr^+^ and n-doped N2200. Energetic analysis reveals that the n-doped species [Mes-Acr^+^···N2200^•−^] is around 9.9 kcal/mol more stable than the undoped neutral complex [Mes-Acr^•^···N2200]. Hirshfeld charge analysis of the [Mes-Acr^+^···N2200^•−^] complex confirms the presence of n-doped N2200, showing charges of 0.9 and −0.9 localized in the Mes-Acr^+^ and N2200^•−^ fragments, respectively, in the acetonitrile solution. The small energy difference between the neutral [Mes-Acr^•^···N2200] and the n-doped species [Mes-Acr^+^···N2200^•−^] implies that both electron configurations can be populated at room temperature.

The reaction that connects the neutral [Mes-Acr^•^···N2200] and the n-doped [Mes-Acr^+^···N2200^•−^] species is the electron transfer process. Because there is no photon involved in the electron transfer step, the reaction is assumed to proceed on the ground state potential energy surface. The ground-state electron transfer from Mes-Acr^•^ to N2200 can occur via two potential mechanisms: ultrafast direct transfer without a barrier or tunneling via a through-space barrier. Real-time TDDFT electronic dynamics demonstrate that charges on the [Mes-Acr^•^···N2200] complex do not exhibit any notable electron transfer within the simulated 50-fs ultrafast timescale (see the Supplementary Materials, fig. S9), suggesting the existence of a tunneling barrier between the nonbonded Mes-Acr^•^ and N2200.

Last, calculations reveal that the formation of the charge-transfer complex [Mes-Acr^+^···N2200^•−^] during the n-doping step does not require a second photon, supporting the one-photon mechanism (Fig. 3A).

### Diffusion-controlled dip doping

**Fig. 4.**
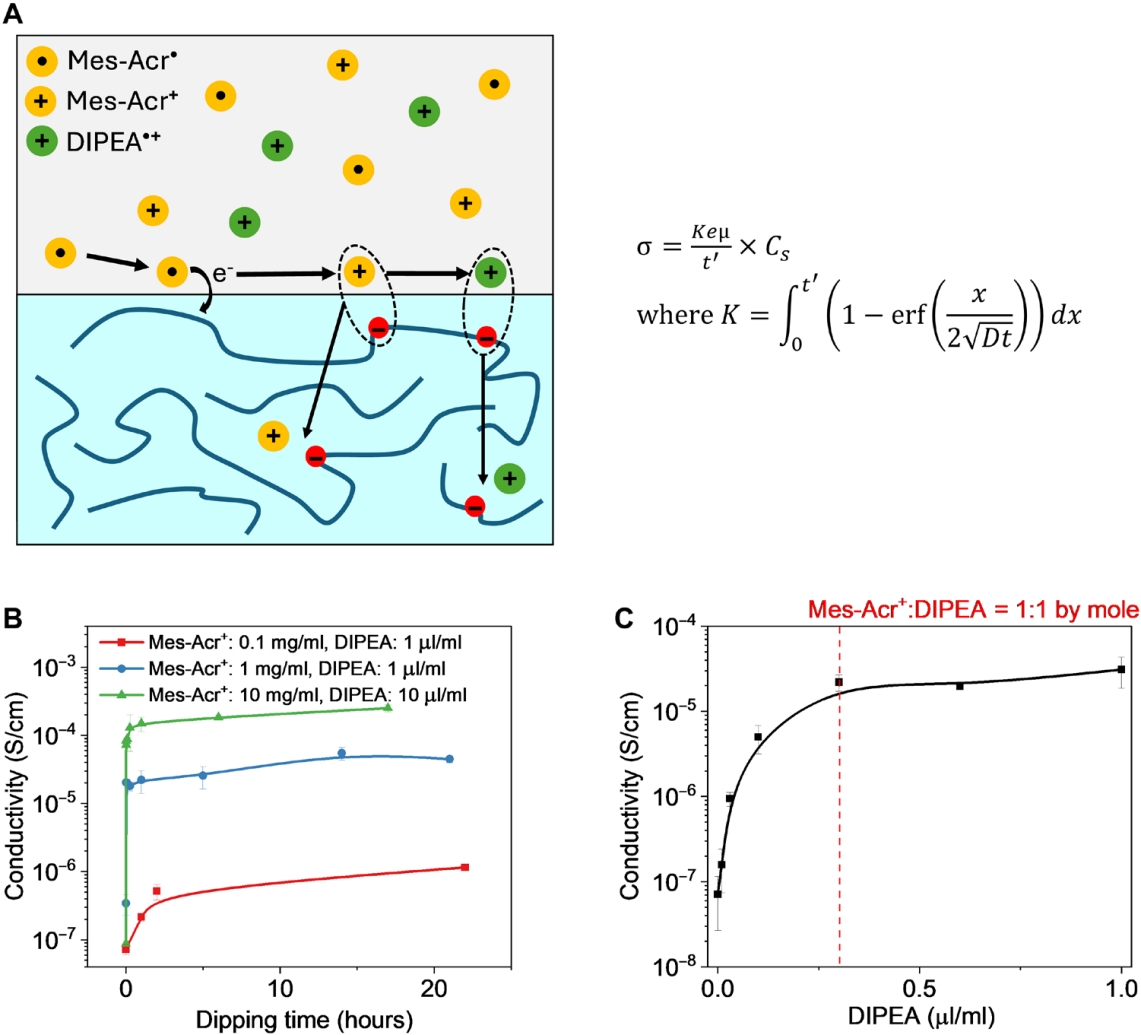
Diffusion-controlled photoredox catalyst–based n-type doping process. (**A**) Schematics of the photoredox catalyst–based n-type doping process where Mes-Acr^•^ needs to diffuse into the conjugated polymer film to enable the doping; given the multiple species in the solution, multiple diffusion events can occur. In theory, Mes-Acr^+^ (after Mes-Acr^•^ shuttles electron to the conjugated polymer) needs to diffuse out of the conjugated polymer film to drive the next catalytic cycle (i.e., forming new Mes-Acr^•^). Derivation of the conductivity equation is detailed in the Supplementary Materials, where *C_s_* is the initial radical concentration in solution, *t*′ is the thickness of the film, μ is the mobility, *e* is the elemental charge, *D* is the diffusion constant, *t* is the diffusion time, and erf is the Gaussian error function. (**B**) Time dependence of conductivity of the doped N2200 film with different amounts of Mes-Acr^+^. (**C**) The conductivity dependence of the doped N2200 film on the starting concentration of DIPEA when Mes-Acr^+^ was in excess (at 1 mg/ml). Note that the molar equivalence of DIPEA at this condition is at ~0.3 μl/ml. (B) and (C) were conducted under the UV-before-dipping condition (i.e., 1 hour illumination of the dopant solution before dipping) and no ionic liquid was used here.

The dip doping process, illustrated in Fig. 1A, relies on the diffusion of the actual dopant (i.e., Mes-Acr^•^ according to Fig. 3A) into the thin film of conjugated polymers. The entire diffusion-controlled doping process (Fig. 4A) consists of the following four plausible steps (under UV light during the doping) based on the doping mechanism (Fig. 3A)

$$\begin{array}{c} \text{Excitation}:\text{Mes}‐{\text{Acr}}^{+}+\text{hv}\overset{k_{\text{ex}}}{\to }\text{Mes}‐{\text{Acr}}^{{+}^{*}} \end{array}$$ (1)

$$\begin{array}{c} \text{Emission}:\text{Mes}‐{\text{Acr}}^{{+}^{*}}\overset{k_{\text{em}}}{\to }\text{Mes}‐{\text{Acr}}^{+}+{\text{hv}}^{'} \end{array}$$ (2)

$$\begin{array}{c} \text{Eletron transfer}:\text{Mes}‐{\text{Acr}}^{{+}^{*}}+\text{DIPEA}\overset{k_{\text{tr}}}{\to }\text{Mes}‐{\text{Acr}}^{•}+{\text{DIPEA}}^{•+} \end{array}$$ (3)

$$\begin{array}{c} \text{Doping}:\text{Mes}‐{\text{Acr}}^{•}+N2200\overset{k_{d}}{\to }\text{Mes}‐{\text{Acr}}^{+}+N2200^{•-} \end{array}$$ (4)

Step (3) indicates that the concentration of Mes-Acr^•^ is dependent on the concentrations of Mes-Acr^+*^ and DIPEA. In theory, the concentration of Mes-Acr^+*^ is affected by multiple factors, including the starting concentration of Mes-Acr^+^, the light intensity and the illumination time [step (1)], as well as the possible decay of Mes-Acr^+*^ to the ground state [step (2)]. Step (4) is a solid-liquid interface reaction where diffusion likely dominates. To focus on the diffusion process of the proximal dopant without complications from concurrent steps (1) through (4) under UV illumination and potential UV-induced degradation of conjugated polymers, we applied UV illumination before doping (i.e., UV before dipping as we discussed earlier). This approach can ensure the completion of steps (1) through (3) and generate a fixed concentration of Mes-Acr^•^, allowing us to focus on the diffusion step. In addition, the presence of the ionic liquid in the earlier concentration dependent experiments (Fig. 3B) could complicate our focus on the diffusion of the actual dopant (i.e., Mes-Acr^•^). Thus, in the following experiments, we only used Mes-Acr^+^ and DIPEA in the doping solution.

Because only a small amount of Mes-Acr^•^ is needed to dope the entire polymer film (assuming 100% doping efficiency, see the Supplementary Materials for calculation), the concentration of Mes-Acr^•^ in the solution can be assumed to remain constant during the diffusion process, which should be proportional to the concentration of Mes-Acr^+^ predetermined (e.g., 0.1 mg/ml, with excess amount of DIPEA). Under these assumptions, we applied the diffusion model of a semi-infinite solid with a constant surface concentration and used Fick’s second law to describe the concentration profile of the diffused species (i.e., Mes-Acr^•^ in our case). To simplify the modeling, we also assumed that both the morphology and the charge/polaron mobility of the N2200 film remain unchanged; thus, the conductivity of the doped N2200 film should theoretically be proportional to the initial concentration of Mes-Acr^•^ (or the concentration of Mes-Acr^+^, under the above assumption). The mathematical derivation of this model is detailed in the Supplementary Materials. Figure 4B shows the fits of the model to the experimental data, showing good agreement particularly under high concentration of Mes-Acr^+^. Notably, as the Mes-Acr^+^ concentration increases from 1 to 10 mg/ml, the measured conductivity is also increased by 10-fold. In another regime, the concentration of DIPEA can become the limiting factor, for example, when the concentration of Mes-Acr^+^ was fixed at 1 mg/ml and the DIPEA concentration was varied from 0.002 to 1 μl/ml (0.0067 to 3.3 relative to Mes-Acr^+^). In this scenario, the conductivity should positively correlate with the concentration of DIPEA initially and then reach a plateau once the amount of DIPEA surpasses the amount of Mes-Acr^+^ and is no longer the limiting reagent. Figure 4C clearly shows this behavior. These data further support the doping mechanism (Fig. 3A).

## DISCUSSION

We have demonstrated facile one-photon–one-electron catalytic photoredox doping of n-type conjugated polymers. We achieved this approach by photoexcitation of an air-stable photoredox catalyst (Mes-Acr^+^BF_4_^−^) that can efficiently transfer electrons to common n-type conjugated polymers via an air-stable weak dopant (amine) in a simple dipping process. Under the photoredox condition, we achieved conductivities of doped n-type conjugated polymer equivalent to values achieved by other less air-stable dopants or under unconventional conditions (*22*). This photoredox catalyst–based n-type doping is facile to operate, applicable to a range of n-type conjugated polymers, and allows control over doping levels via multiple means. Given the mild process, catalytic nature, and numerous photoredox catalysts with a wide range of redox potentials (*17*, *30*), the photoredox catalyst–based doping will find numerous applications and thereby warrant intensive research in the future.

## MATERIALS AND METHODS

### Materials

N2200 was purchased from 1-Material. Mes-Acr^+^BF_4_^−^, acetonitrile, BBL, and DIPEA were purchased from Sigma-Aldrich. 4,9-Dibromo-2,7-bis(2-octyldodecyl)benzo[lmn][3,8]phenanthroline-1,3,6,8(2*H*,7*H*)-tetraone was purchased from SunaTech. All materials were used as received without any further treatment. High-temperature gel permeation chromatography (HT-GPC) measurements were performed on an Agilent 1260 HT-GPC instrument with TCB (1,2,4-trichlorobenzene) as the eluent at 140°C. The obtained molar mass is relative to the polystyrene standard. ^1^H nuclear magnetic resonance (NMR) measurements were recorded with a Bruker DRX spectrometer (400 MHz).

### CV measurement

CV measurements were conducted using a CH Instruments 600E potentiostat with a standard three-electrode configuration. A three-electrode cell of a glassy carbon working electrode, Ag/Ag^+^ reference electrode, and Pt counter electrode were used. A solution (0.1 M) of tetrabutylammonium hexafluorophosphate in anhydrous acetonitrile was used as a supporting electrolyte. The reference electrode was calibrated using a ferrocene/ferrocenium redox couple. Mes-Acr^+^BF_4_^−^ was measured in solution and polymers as thin films. eV = −[5.09 +E(V)].

### Preparation of conjugated polymer films

The glass substrates (1.5 cm by 1.5 cm) were precleaned by sequential sonication for 15 min each in deionized (DI) water with detergent, DI water (twice), acetone, and isopropanol. The substrates were then dried under nitrogen gas flow and cleaned using UV-ozone treatment for 15 min. Subsequently, they were transferred to a nitrogen-filled glovebox for further use.

For the preparation of N2200, PNDI-g2-2T, and N2200-OEG films, these polymers were dissolved in chloroform at a concentration of 10 mg/ml. The solution was then spun coated on glass substrates at room temperature at 1000 rpm for 60 s to obtain ~100-nm-thick films.

By contrast, BBL was dissolved in methanesulfonic acid (MSA) at the same concentration of 10 mg/ml at 70°C to ensure complete dissolution. The solution was then spun cast on glass substrate at 2000 rpm for 60 s. The obtained BBL films were immersed in methanol for 30 s and then dried under a flow of nitrogen gas. This step was repeated three times to remove any residual MSA. The films were then soaked in DI water overnight to further remove any residual MSA. Last, the films were dried in a vacuum oven at 60°C for 6 hours to remove any remaining water.

### Photoredox catalyst dip doping

Typically, 1.5 mg of Mes-Acr^+^BF_4_^−^and 1.5 μl of DIPEA were dissolved in 1.5 ml of acetonitrile in a 20-ml sample vial.

#### UV during dipping

The coated conjugated polymer film was dipped into the above solution of Mes-Acr^+^BF_4_^−^ and DIPEA. The cap was tightened on the vial and the UV light (Blak-Ray B-100A high-intensity UV lamp, 100 W, 365 nm) was turned on to illuminate the film and the solution (from outside the vial). After the set illumination time, the UV lamp was turned off and the film was taken out of the solution and dried by nitrogen gas flow.

#### UV before dipping

The cap was tightened on the vial containing the Mes-Acr^+^BF_4_^−^ and DIPEA solution and the UV light was turned on to illuminate the solution. The solution color turned from yellow to red in several minutes. After the set illumination time (for instance, 1 hour), the UV lamp was turned off and the coated semiconducting polymer film was dipped into the above illuminated solution of Mes-Acr^+^BF_4_^−^ and DIPEA. After the desired dipping time, the film was taken out of the solution and dried by nitrogen gas flow.

### Conductivity measurement

The doped polymer films were transferred into a thermal evaporator to evaporate 80 nm Al at a rate of ~1.4 Å/s with a shadow mask. The channel length is 1 mm and the channel width is 9 mm. The *I*-*V* response was measured with a Kethley 2400 source meter in two-probe mode. The conductivity was calculated as below

$$\sigma =\left(I/V\right)\times \frac{d}{\mathrm{wl}}$$

where *I/V* is the slope of the *I*-*V* curve, *d* is the channel length (1 mm), *l* is the film thickness, and *w* is the channel width (9 mm). The average value of four devices was shown and the standard deviation was used as error bar.

### UV photoelectron spectroscopy

The UPS was performed with a Kratos Axis Supra x-ray photoelectron spectrometer. N2200 films were spun coated on Si substrate and were doped as previously mentioned. Then, the samples were sealed into plastic bags inside the glovebox of nitrogen gas and transferred to the UPS chamber for the UPS characterization.

### Electron paramagnetic resonance

The EPR was performed with a JEOL X-Band EPR. The polymer was coated on poly(ethylene terephthalate) (PET) substrate (15 mm by 15 mm) and doped as previously mentioned. Then, the PET was cut into 3 mm by 15 mm pieces and put into an NMR tube and sealed inside the glovebox. The sample was then transferred to the EPR chamber for measurement.

### Frequency-modulated scanning kelvin probe microscopy

Frequency-modulated scanning kelvin probe microscopy (FM-SKPM) was performed using an Oxford Instruments MFP3D-Bio atomic force microscope. The FM-SKPM measurements were performed using custom-written Igor code (code available online: https://github.com/GingerLabUW/AFMSoftware), with an additional external lock-in amplifier. Images were taken under flowing nitrogen in a sealed flow cell under dark conditions, and the flow cell and samples were loaded in an inert nitrogen environment glovebox. Atomic Force Microscopy measurements were performed using Pt-coated cantilevers with resonance frequency ~300 kHz and spring constant ~40 N/m.

## Supplementary Materials

### This PDF file includes:

Supplementary Materials and Methods Figs. S1 to S13 Tables S1 and S2 Notes S1 and S2 Supplementary Text References

## References

[1] A. D. Scaccabarozzi, A. Basu, F. Aniés, J. Liu, O. Zapata-Arteaga, R. Warren, Y. Firdaus, M. I. Nugraha, Y. Lin, M. Campoy-Quiles, N. Koch, C. Müller, L. Tsetseris, M. Heeney, T. D. Anthopoulos, Doping approaches for organic semiconductors. Chem. Rev. 122, 4420–4492 (2022). 34793134 10.1021/acs.chemrev.1c00581

[2] S. R. Marder, S. Barlow, Reaction mechanisms for electrical doping of organic semiconductors using complex dopants. Chem. Phys. Rev. 5, 021303 (2024).

[3] C. G. Tang, M. N. Syafiqah, Q.-M. Koh, C. Zhao, J. Zaini, Q.-J. Seah, M. J. Cass, M. J. Humphries, I. Grizzi, J. H. Burroughes, R.-Q. Png, L.-L. Chua, P. K. H. Ho, Multivalent anions as universal latent electron donors. Nature 573, 519–525 (2019). 31554981 10.1038/s41586-019-1575-7

[4] S.-G. Kim, G. C. Fish, E. Socie, A. T. Terpstra, D.-A. Park, K. Zhu, M. Grätzel, J.-E. Moser, N.-G. Park, Photo-doping of spiro-OMeTAD for highly stable and efficient perovskite solar cells. Joule 8, 1707–1722 (2024).

[5] T. Zhang, F. Wang, H.-B. Kim, I.-W. Choi, C. Wang, E. Cho, R. Konefal, Y. Puttisong, K. Terado, L. Kobera, M. Chen, M. Yang, S. Bai, B. Yang, J. Suo, S.-C. Yang, X. Liu, F. Fu, H. Yoshida, W. M. Chen, J. Brus, V. Coropceanu, A. Hagfeldt, J.-L. Brédas, M. Fahlman, D. S. Kim, Z. Hu, F. Gao, Ion-modulated radical doping of spiro-OMeTAD for more efficient and stable perovskite solar cells. Science 377, 495–501 (2022). 35901165 10.1126/science.abo2757

[6] N. Sakai, R. Warren, F. Zhang, S. Nayak, J. Liu, S. V. Kesava, Y.-H. Lin, H. S. Biswal, X. Lin, C. Grovenor, T. Malinauskas, A. Basu, T. D. Anthopoulos, V. Getautis, A. Kahn, M. Riede, P. K. Nayak, H. J. Snaith, Adduct-based p-doping of organic semiconductors. Nat. Mater. 20, 1248–1254 (2021). 33888905 10.1038/s41563-021-00980-x

[7] J. Liu, B. van der Zee, R. Alessandri, S. Sami, J. Dong, M. I. Nugraha, A. J. Barker, S. Rousseva, L. Qiu, X. Qiu, N. Klasen, R. C. Chiechi, D. Baran, M. Caironi, T. D. Anthopoulos, G. Portale, R. W. A. Havenith, S. J. Marrink, J. C. Hummelen, L. J. A. Koster, N-type organic thermoelectrics: Demonstration of ZT > 0.3. Nat. Commun. 11, 5694 (2020). 33173050 10.1038/s41467-020-19537-8 PMC7655812

[8] B. Russ, A. Glaudell, J. J. Urban, M. L. Chabinyc, R. A. Segalman, Organic thermoelectric materials for energy harvesting and temperature control. Nat. Rev. Mater. 1, 16050 (2016).

[9] X. Yan, M. Xiong, X.-Y. Deng, K.-K. Liu, J.-T. Li, X.-Q. Wang, S. Zhang, N. Prine, Z. Zhang, W. Huang, Y. Wang, J.-Y. Wang, X. Gu, S. K. So, J. Zhu, T. Lei, Approaching disorder-tolerant semiconducting polymers. Nat. Commun. 12, 5723 (2021). 34588457 10.1038/s41467-021-26043-y PMC8481336

[10] X. Lin, B. Wegner, K. M. Lee, M. A. Fusella, F. Zhang, K. Moudgil, B. P. Rand, S. Barlow, S. R. Marder, N. Koch, A. Kahn, Beating the thermodynamic limit with photo-activation of n-doping in organic semiconductors. Nat. Mater. 16, 1209–1215 (2017). 29170548 10.1038/nmat5027

[11] B. D. Naab, S. Guo, S. Olthof, E. G. B. Evans, P. Wei, G. L. Millhauser, A. Kahn, S. Barlow, S. R. Marder, Z. Bao, Mechanistic study on the solution-phase n-doping of 1,3-dimethyl-2-aryl-2,3-dihydro-1H-benzoimidazole derivatives. J. Am. Chem. Soc. 135, 15018–15025 (2013). 24011269 10.1021/ja403906d PMC3994987

[12] X. Zhao, D. Madan, Y. Cheng, J. Zhou, H. Li, S. M. Thon, A. E. Bragg, M. E. DeCoster, P. E. Hopkins, H. E. Katz, High conductivity and electron-transfer validation in an n-type fluoride-anion-doped polymer for thermoelectrics in air. Adv. Mater. 29, 1606928 (2017). 10.1002/adma.201606928 28707300

[13] O. Bardagot, C. Aumaître, A. Monmagnon, J. Pécaut, P.-A. Bayle, R. Demadrille, Revisiting doping mechanisms of n-type organic materials with N-DMBI for thermoelectric applications: Photo-activation, thermal activation, and air stability. Appl. Phys. Lett. 118, 203904 (2021).

[14] A. Tlili, S. Lakhdar, Acridinium salts and cyanoarenes as powerful photocatalysts: Opportunities in organic synthesis. Angew. Chem. Int. Ed. Engl. 60, 19526–19549 (2021). 33881207 10.1002/anie.202102262

[15] M. Melchionna, P. Fornasiero, Updates on the roadmap for photocatalysis. ACS Catal. 10, 5493–5501 (2020).

[16] C. K. Prier, D. A. Rankic, D. W. C. MacMillan, Visible light photoredox catalysis with transition metal complexes: Applications in organic synthesis. Chem. Rev. 113, 5322–5363 (2013). 23509883 10.1021/cr300503r PMC4028850

[17] N. A. Romero, D. A. Nicewicz, Organic photoredox catalysis. Chem. Rev. 116, 10075–10166 (2016). 27285582 10.1021/acs.chemrev.6b00057

[18] I. A. MacKenzie, L. Wang, N. P. R. Onuska, O. F. Williams, K. Begam, A. M. Moran, B. D. Dunietz, D. A. Nicewicz, Discovery and characterization of an acridine radical photoreductant. Nature 580, 76–80 (2020). 32238940 10.1038/s41586-020-2131-1 PMC7138348

[19] W. Jin, C.-Y. Yang, R. Pau, Q. Wang, E. K. Tekelenburg, H.-Y. Wu, Z. Wu, S. Y. Jeong, F. Pitzalis, T. Liu, Q. He, Q. Li, J.-D. Huang, R. Kroon, M. Heeney, H. Y. Woo, A. Mura, A. Motta, A. Facchetti, M. Fahlman, M. A. Loi, S. Fabiano, Photocatalytic doping of organic semiconductors. Nature 630, 96–101 (2024). 38750361 10.1038/s41586-024-07400-5 PMC11153156

[20] D. Yuan, W. Liu, X. Zhu, Efficient and air-stable n-type doping in organic semiconductors. Chem. Soc. Rev. 52, 3842–3872 (2023). 37183967 10.1039/d2cs01027e

[21] S. Griggs, A. Marks, H. Bristow, I. McCulloch, n-Type organic semiconducting polymers: Stability limitations, design considerations and applications. J. Mater. Chem. C 9, 8099–8128 (2021). 10.1039/d1tc02048j PMC8264852 34277009

[22] H. Guo, C.-Y. Yang, X. Zhang, A. Motta, K. Feng, Y. Xia, Y. Shi, Z. Wu, K. Yang, J. Chen, Q. Liao, Y. Tang, H. Sun, H. Y. Woo, S. Fabiano, A. Facchetti, X. Guo, Transition metal-catalysed molecular n-doping of organic semiconductors. Nature 599, 67–73 (2021). 34732866 10.1038/s41586-021-03942-0

[23] Y. Yamashita, J. Tsurumi, M. Ohno, R. Fujimoto, S. Kumagai, T. Kurosawa, T. Okamoto, J. Takeya, S. Watanabe, Efficient molecular doping of polymeric semiconductors driven by anion exchange. Nature 572, 634–638 (2019). 31462795 10.1038/s41586-019-1504-9

[24] R. A. Schlitz, F. G. Brunetti, A. M. Glaudell, P. L. Miller, M. A. Brady, C. J. Takacs, C. J. Hawker, M. L. Chabinyc, Solubility-limited extrinsic n-type doping of a high electron mobility polymer for thermoelectric applications. Adv. Mater. 26, 2825–2830 (2014). 24448874 10.1002/adma.201304866

[25] G. Ye, J. Liu, X. Qiu, S. Stäter, L. Qiu, Y. Liu, X. Yang, R. Hildner, L. J. A. Koster, R. C. Chiechi, Controlling n-type molecular doping via regiochemistry and polarity of pendant groups on low band gap donor–acceptor copolymers. Macromolecules 54, 3886–3896 (2021). 34054145 10.1021/acs.macromol.1c00317 PMC8154869

[26] J. Liu, L. Qiu, R. Alessandri, X. Qiu, G. Portale, J. Dong, W. Talsma, G. Ye, A. A. Sengrian, P. C. T. Souza, M. A. Loi, R. C. Chiechi, S. J. Marrink, J. C. Hummelen, L. J. A. Koster, Enhancing molecular n-type doping of donor–acceptor copolymers by tailoring side chains. Adv. Mater. 30, 1704630 (2018). 10.1002/adma.201704630 29325212

[27] D. Rosas Villalva, S. Singh, L. A. Galuska, A. Sharma, J. Han, J. Liu, M. A. Haque, S. Jang, A. H. Emwas, L. J. A. Koster, X. Gu, B. C. Schroeder, D. Baran, Backbone-driven host–dopant miscibility modulates molecular doping in NDI conjugated polymers. Mater. Horiz. 9, 500–508 (2022). 34927646 10.1039/d1mh01357b PMC8725799

[28] A. Babel, S. A. Jenekhe, High electron mobility in ladder polymer field-effect transistors. J. Am. Chem. Soc. 125, 13656–13657 (2003). 14599192 10.1021/ja0371810

[29] J. Guo, L. Q. Flagg, D. K. Tran, S. E. Chen, R. Li, N. B. Kolhe, R. Giridharagopal, S. A. Jenekhe, L. J. Richter, D. S. Ginger, Hydration of a side-chain-free n-type semiconducting ladder polymer driven by electrochemical doping. J. Am. Chem. Soc. 145, 1866–1876 (2023). 36630664 10.1021/jacs.2c11468

[30] N. Holmberg-Douglas, D. A. Nicewicz, Photoredox-catalyzed C–H functionalization reactions. Chem. Rev. 122, 1925–2016 (2022). 34585909 10.1021/acs.chemrev.1c00311 PMC8939264

[31] P. Hohenberg, W. Kohn, Inhomogeneous electron gas. Phys. Rev. 136, B864–B871 (1964).

[32] W. Kohn, L. J. Sham, Self-consistent equations including exchange and correlation effects. Phys. Rev. 140, A1133–A1138 (1965).

[33] E. Runge, E. K. U. Gross, Density-functional theory for time-dependent systems. Phys. Rev. Lett. 52, 997–1000 (1984).

[34] J. P. Perdew, K. Burke, M. Ernzerhof, Generalized gradient approximation made simple. Phys. Rev. Lett. 77, 3865–3868 (1996). 10062328 10.1103/PhysRevLett.77.3865

[35] C. Adamo, V. Barone, Toward reliable density functional methods without adjustable parameters: The PBE0 model. J. Chem. Phys. 110, 6158–6170 (1999).

[36] M. J. Frisch, G. W. Trucks, H. B. Schlegel, G. E. Scuseria, M. A. Robb, J. R. Cheeseman, G. Scalmani, V. Barone, G. A. Petersson, H. Nakatsuji, X. Li, M. Caricato, A. V. Marenich, J. Bloino, B. G. Janesko, R. Gomperts, B. Mennucci, H. P. Hratchian, J. V. Ortiz, A. F. Izmaylov, J. L. Sonnenberg, Williams, F. Ding, F. Lipparini, F. Egidi, J. Goings, B. Peng, A. Petrone, T. Henderson, D. Ranasinghe, V. G. Zakrzewski, J. Gao, N. Rega, G. Zheng, W. Liang, M. Hada, M. Ehara, K. Toyota, R. Fukuda, J. Hasegawa, M. Ishida, T. Nakajima, Y. Honda, O. Kitao, H. Nakai, T. Vreven, K. Throssell, J. A. Montgomery Jr., J. E. Peralta, F. Ogliaro, M. J. Bearpark, J. J. Heyd, E. N. Brothers, K. N. Kudin, V. N. Staroverov, T. A. Keith, R. Kobayashi, J. Normand, K. Raghavachari, A. P. Rendell, J. C. Burant, S. S. Iyengar, J. Tomasi, M. Cossi, J. M. Millam, M. Klene, C. Adamo, R. Cammi, J. W. Ochterski, R. L. Martin, K. Morokuma, O. Farkas, J. B. Foresman, D. J. Fox, Gaussian 16 Rev. J.14+. Wallingford, CT (2020).

[37] S. Grimme, S. Ehrlich, L. Goerigk, Effect of the damping function in dispersion corrected density functional theory. J. Comput. Chem. 32, 1456–1465 (2011). 21370243 10.1002/jcc.21759

[38] F. Weigend, R. Ahlrichs, Balanced basis sets of split valence, triple zeta valence and quadruple zeta valence quality for H to Rn: Design and assessment of accuracy. Phys. Chem. Chem. Phys. 7, 3297–3305 (2005). 16240044 10.1039/b508541a

[39] F. Weigend, M. Häser, H. Patzelt, R. Ahlrichs, RI-MP2: Optimized auxiliary basis sets and demonstration of efficiency. Chem. Phys. Lett. 294, 143–152 (1998).

[40] A. V. Marenich, C. J. Cramer, D. G. Truhlar, Universal solvation model based on solute electron density and on a continuum model of the solvent defined by the bulk dielectric constant and atomic surface tensions. J. Phys. Chem. B. 113, 6378–6396 (2009). 19366259 10.1021/jp810292n

[41] J. Tomasi, B. Mennucci, R. Cammi, Quantum mechanical continuum solvation models. Chem. Rev. 105, 2999–3094 (2005). 16092826 10.1021/cr9904009

[42] W. Liang, C. T. Chapman, X. Li, Efficient first-principles electronic dynamics. J. Chem. Phys. 134, 184102 (2011). 21568492 10.1063/1.3589144

[43] D. B. Williams-Young, A. Petrone, S. Sun, T. F. Stetina, P. Lestrange, C. E. Hoyer, D. R. Nascimento, L. Koulias, A. Wildman, J. Kasper, J. J. Goings, F. Ding, A. E. DePrince III, E. F. Valeev, X. Li, The Chronus Quantum software package. WIREs Comput. Mol. Sci. 10, e1436 (2020).

[44] P. D. Nguyen, F. Ding, S. A. Fischer, W. Liang, X. Li, Solvated first-principles excited-state charge-transfer dynamics with time-dependent polarizable continuum model and solvent dielectric relaxation. J. Phys. Chem. Lett. 3, 2898–2904 (2012).

[45] X. Zhao, M. Alsufyani, J. Tian, Y. Lin, S. Y. Jeong, H. Y. Woo, Y. Yin, I. McCulloch, High efficiency n-type doping of organic semiconductors by cation exchange. Adv. Mater. 36, e2412811 (2024). 39385648 10.1002/adma.202412811

[46] J. Song, Y. Li, Y. Cai, R. Zhang, S. Wang, J. Xin, L. Han, D. Wei, W. Ma, F. Gao, Y. Sun, Solid additive engineering enables high-efficiency and eco-friendly all-polymer solar cells. Matter 5, 4047–4059 (2022).

[47] “Diffusion in dilute solutions,” in *Diffusion: Mass Transfer in Fluid Systems*, E. L. Cussler, Ed., Cambridge Series in Chemical Engineering (Cambridge Univ. Press, ed. 3, 2009), pp. 13–55.

