# Supplementary material for: Air-stable n-type dopant for organic semiconductors via a single-photon catalytic process
Source: Sci Adv. 2025 Jun 6;11(23):eadu8215. doi: 10.1126/sciadv.adu8215 (PMC12143396; doi:10.1126/sciadv.adu8215)
Supplement: Supplementary file 1 — Supplementary Materials and Methods Figs. S1 to S13 Tables S1 and S2 Notes S1 and S2 Supplementary Text References [file sciadv.adu8215_sm.pdf]

Science Advances
AAAS

Supplementary Materials for

Air-stable n-type dopant for organic semiconductors via a single-photon catalytic process

Liang Yan *et al.*

Corresponding author: Wei You, wyou@unc.edu

Sci. Adv. **11**, eadu8215 (2025)
DOI: 10.1126/sciadv.adu8215

This PDF file includes:

Supplementary Materials and Methods
Figs. S1 to S13
Tables S1 and S2
Notes S1 and S2
Supplementary Text
References

Materials and Methods

Synthesis n-type semiconducting polymers:

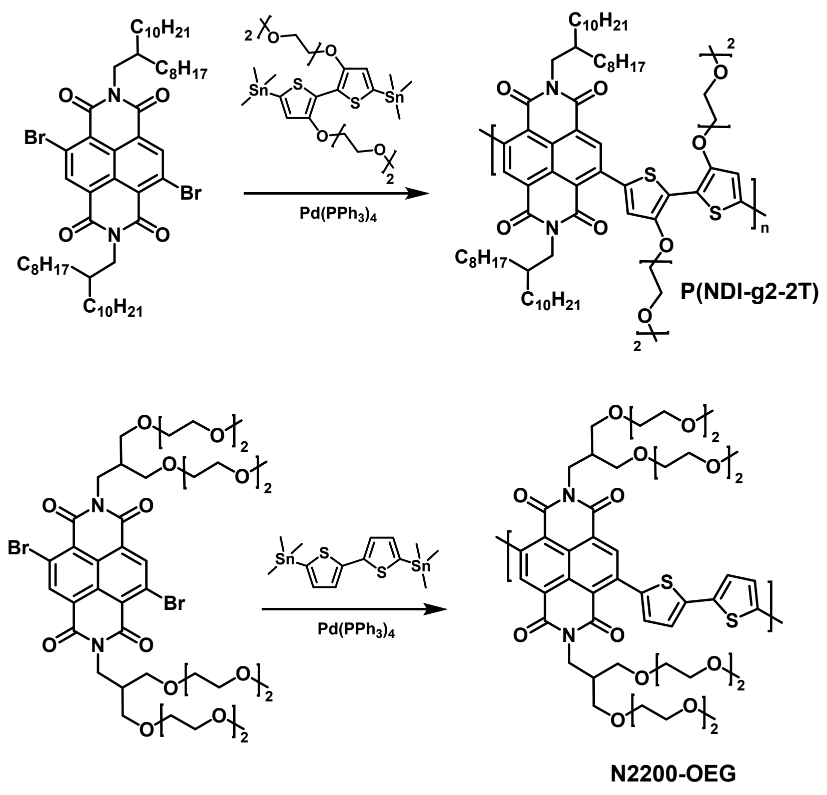

Fig. S1.

Synthesis of P(NDI-g2-2T) and N2200-OEG.

diBromo-OEG-NDI: 4,9-dibromo-2,7-bis(3-(2-(2-methoxyethoxy)ethoxy)-2-((2-(2-methoxyethoxy)ethoxy)methyl)propyl)benzo[lmn][3,8]phenanthroline-1,3,6,8(2H,7H)-tetraone was synthesized according to previous literature.[2] ^{1}H NMR (400 MHz, CDCl3) δ 8.97 (s, 1H), 4.33 (d, J = 7.0 Hz, 2H), 3.63 – 3.42 (m, 20H), 3.33 (s, 6H), 2.54 (p, J = 6.3 Hz, 1H).

P(NDI-g2-2T): P(NDI-g2-2T) was polymerized according to previous literature.[1] M_n: 33k, Đ: 1.8, determined by GPC in TCB at 140 °C.

N2200-OEG: dibromo-OEG-NDI (159.7 mg, 1 Eq, 158.3 μmol), 5,5'-bis(trimethylstannyl)-2,2'-bithiophene (77.9 mg, 1 Eq, 158.3 μmol) and tetrakis(triphenylphosphine)palladium(0) (7.3 mg, 0.04 Eq, 6.333 μmol) were charged into a 10 mL microwave reactor vial. The mixture was evacuated and refilled with argon for three cycles before addition of anhydrous toluene (1.7 mL) and DMF (0.17 mL) under argon stream. The reaction was heated up to 200 °C and held in a CEM microwave reactor (300 W) for 60 min. After the polymerization, the crude polymer was dissolved in hot tetrachloroethane and precipitated into hexanes. The collected polymer was extracted via a Soxhlet extractor with ethyl acetate and collected in chloroform. The polymer solution was concentrated and re-precipitated and collected via filtration and dried under vacuum as a dark solid (135 mg). Mn: 44k, Đ: 2.0 determined by GPC in TCB at 140 °C.

DFT Calculations:

To analyze the underlying reaction pathways, we carried out computational study on the mechanisms of doping N2200 under the photoredox condition. We applied density functional theory (DFT) (*31, 32*) and time-dependent DFT (TDDFT) (*33*) with the hybrid PBE0 functional(*34, 35*) using the Gaussian 16 quantum chemistry program package.(*36*) The D3 version of Grimme's dispersion with Becke-Johnson damping(*37*) was used for the empirical dispersion correction of non-covalent interactions. All-electron 6-31++G(d,p) basis set(*38, 39*) was used in geometry optimization, frequency calculations, and quantum dynamics simulations. Basis set superposition error is account for in the energetic calculations. Numerical integrations of DFT were carried out with an ultrafine grid (99,590). All structures were optimized in the acetonitrile (ε = 35.688) solvent. The solvent effect was calculated using the SMD(*40*) variation of the integral equation formalism polarizable continuum (IEFPCM) solvation model.(*41*) The optimized intermediate and transition state structures were confirmed to have no imaginary frequency and only one imaginary frequency, respectively. Transition states were further verified to connect correct stationary points by intrinsic reaction coordinate (IRC) calculations. Thermal corrections of the structures in the reaction mechanism profile were obtained through harmonic potential approximations at 298.15 K and 1 atm pressure on optimized structures.

Quantum Electronic Dynamics:

The ultrafast charge-transfer between the weakly interacting monomers of [Mes-Acr$^{\bullet}$···N2200] is modeled using the RT-TDDFT (real-time time-dependent density functional theory) (*42*) module in the Chronus Quantum package.(*43*) These simulations incorporate both static and real-time electronic responses to solvation effects through the implementation of a time-dependent polarizable continuum model (TDPCM).(*44*) Considering the femtosecond time-scale for direct charge-transfer dynamics, a smaller basis set, 6-31G(d,p), was utilized to reduce computational

costs while maintaining sufficient accuracy. The acetonitrile solvent is modeled on a coarse grid of 302 points with a bulk dielectric constant $\varepsilon_0 = 35.7$, optical dielectric $\varepsilon_\infty = 1.8$, and relaxation time $\tau = 5.9$ ps. To model the charge-transfer pathways from the electron donor Mes-Acr$^\bullet$ to the N2200 dopant, a static field with an intensity of I = 0.0025 au (1.29 × 10^9 V/m), opposite to the direction of electron motion (i.e., pointing from the N2200 moiety to Mes-Acr$^\bullet$ along their intramolecular axis), was applied during the SCF stage to prepare the neutral state before charge transfer between the monomers. Following the removal of the static field, non-equilibrium electron dynamics are captured by real-time simulations with a time-step $\Delta t = 0.1$ au (~0.0024 fs) up to $t_{max} = 50$ fs. The time-dependent Mulliken charge (**Figure S8**) exhibits small local oscillations without significant charge transfer, suggesting the existence of a tunneling barrier that prevents the direct electron transfer from taking place.

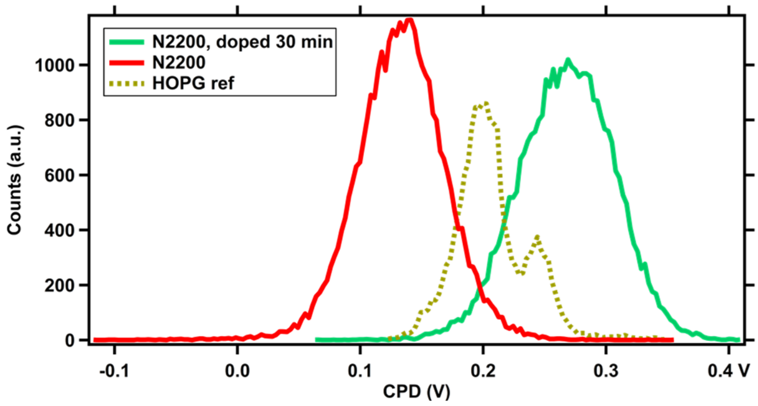

Fig. S2.

Fermi level measured by Kelvin probe microscopy. Contact potential difference (CPD) histograms for the neat film (N2200) and film doped for 30 minutes, as measured with frequency-modulation scanning Kelvin probe microscopy (FM-SKPM). Here, the measurements show the doping of ~130 meV shift in the Fermi level, as indicated by a more negative CPD (deeper work function). The measurements were calibrated against a highly-ordered pyrolytic graphite (HOPG) reference sample (E_f= -4.65 eV) to determine the absolute work function value (undoped N2200 showed E_f= -4.75 eV, 30-min doped N2200 showed E_f= -4.62 eV).

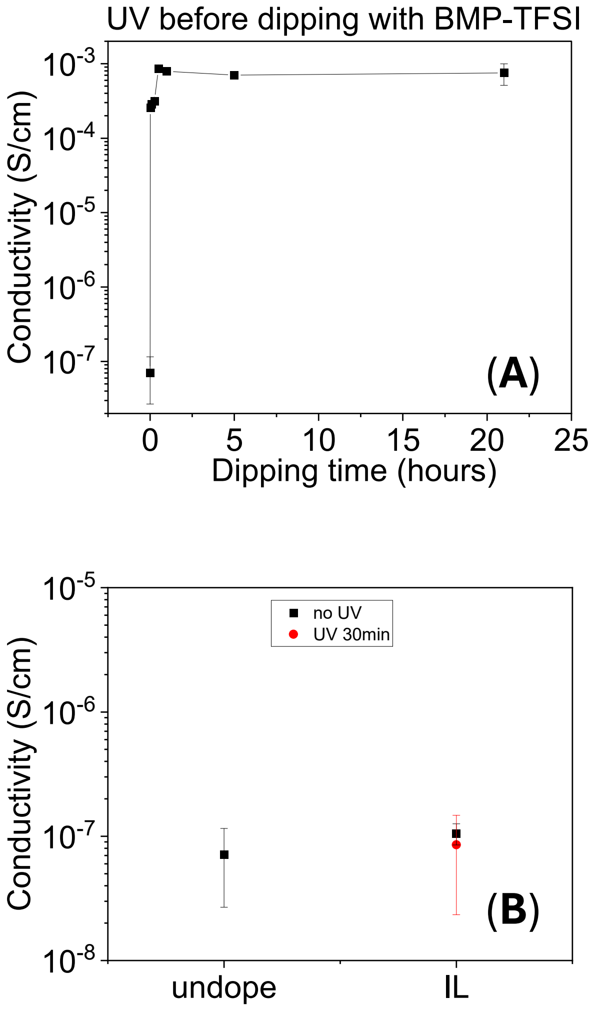

Fig. S3.

BMP-TFSI ion exchange effect on dip doping for N2200. (**A**) Dip doping results with BMP-TFSI ion exchange for N2200 with UV before dipping method (i.e., 1 hour illumination of the dopant solution before dipping). BMP-TFSI was added in the solution containing Mes-$Acr^+BF_4^-$ and DIPEA before UV illumination. BMP-TFSI: Mes-Acr^+=10:1 by mole. (**B**) Dip doping for N2200 in IL (BMP-TFSI) only solution. The BMP-TFSI concentration is the same as the dip doping in (**A**).

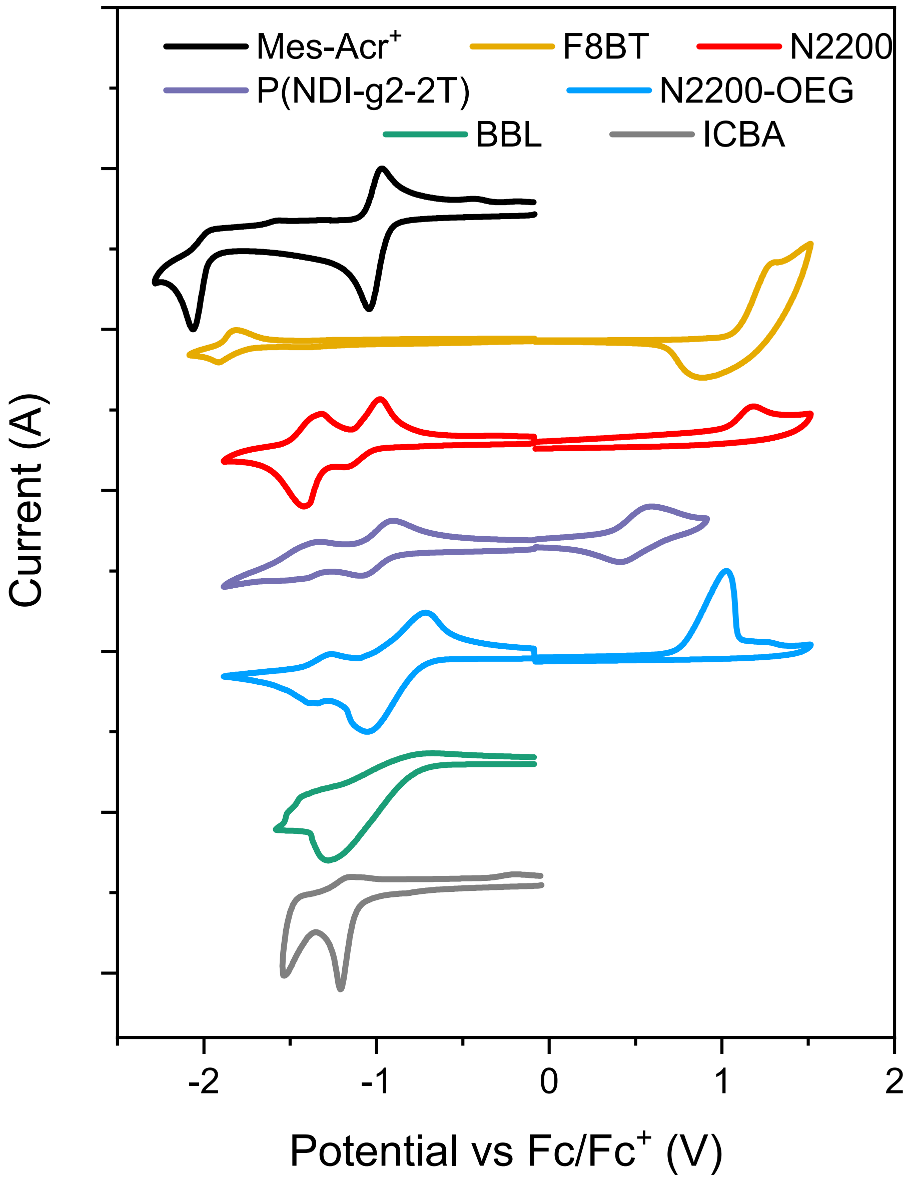

Fig. S4.

CV curves of polymers and photoredox catalyst.

Table S1.

CV data for photocatalyst Mes-$Acr^{+}BF_4^{-}$.

Redox Event	Max (V)	Min (V)	$E_{1/2}$ (V)	[a]eV
1	-0.96	-1.04	-1.00	– 4.09
2	-1.96	-2.06	-2.01	– 3.08

[a]Calculated from the $E_{1/2}$, the 1st redox event is the reduction of Mes-Acr^{+} to Acr-$Mes^{•}$, the 2nd redox event is the reduction of Acr-$Mes^{•}$ to the corresponding anionic species.

Table S2.

CV data for polymers.

Polymer	E_{OX} (V)	E_{RED} (V)	[a]-IP^{CV} (eV)	[b]-EA^{CV} (eV)
F8BT	1.07	– 1.71	– 6.16	– 3.38
N2200	1.01	– 1.02	– 6.10	– 4.07
P(NDI-g2-2T)	0.37	– 0.90	– 5.46	– 4.19
N2200-OEG	0.77	– 0.78	– 5.86	– 4.31
ICBA	-	– 0.77	-	– 3.97
BBL	-	– 1.12	-	– 4.32

[a] Calculated from the E_{OX}. [b] Calculated from the E_{RED}. (Note: eV = -(5.09 + E(V), where Fc/Fc^+ was defined at – 5.09 eV vs. vacuum).

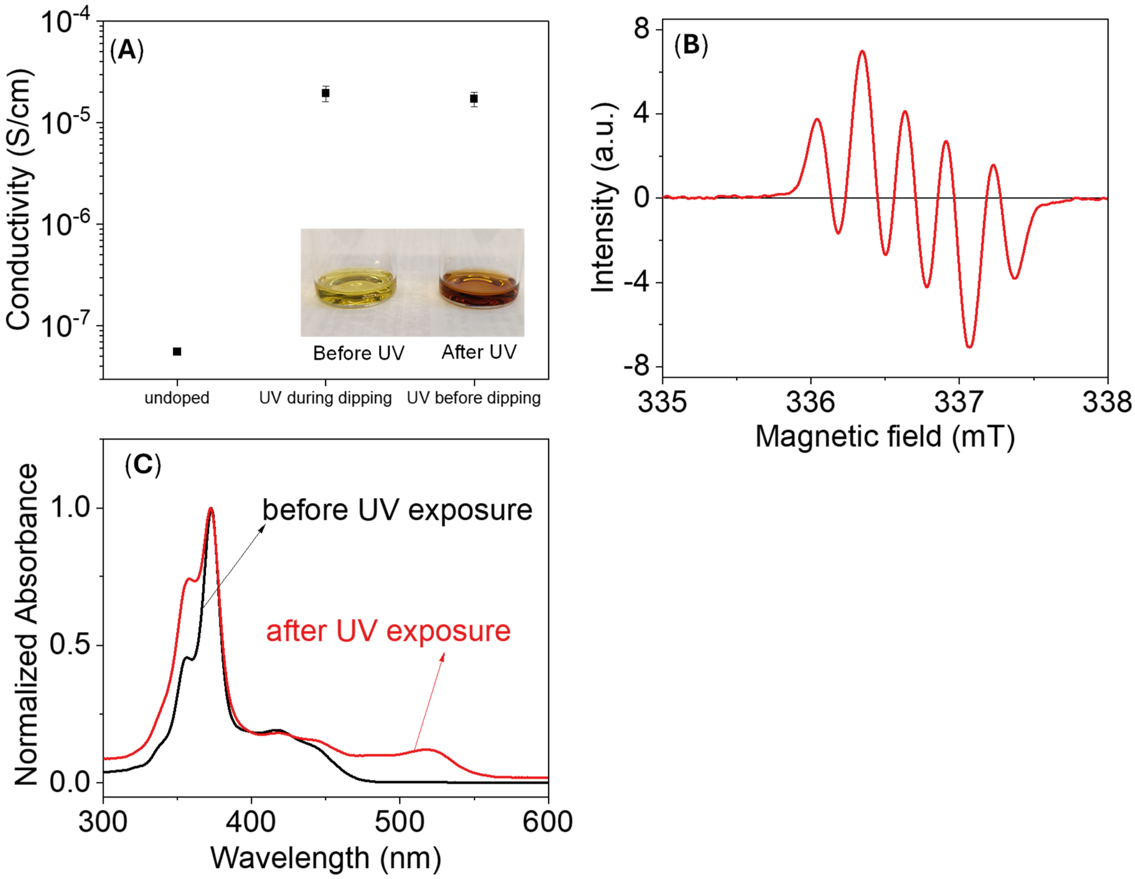

Fig. S5.

Comparison of UV during dipping and UV before dipping. (**A**) Dip doping results for N2200 with UV during dipping and UV before dipping. The dipping time was 15 min for both cases, UV-before dipping started with 1 hour illumination of the dopant solution before dipping.; the inset shows the color change of solution of Mes-Acr$^+$BF$_4^-$ and DIPEA in acetonitrile before UV illumination and after UV illumination. Mes-Acr$^+$BF$_4^-$ concentration is 1 mg/mL and DIPEA concentration is 1 μL/mL. (**B**) EPR signal for acetonitrile solution containing Mes-Acr$^+$BF$_4^-$ (1 mg/mL) and DIPEA (1 μL/mL) after 365 nm UV exposure for 0.5 hour and storage in dark in glove box overnight. The solution was further diluted with *o*-xylene by 1:10 before EPR measurement. The EPR signal is very similar to the Mes-Acr• radical reported in reference *(18)*. (**C**) absorption spectrum of acetonitrile solution containing Mes-Acr$^+$BF$_4^-$ (1 mg/mL) and DIPEA (1 μL/mL) before and after 365 nm UV exposure for 0.5 hours. The solution after UV exposure was further stored in dark in glove box overnight. All solutions were diluted with acetonitrile by 1:30 before absorption measurement.

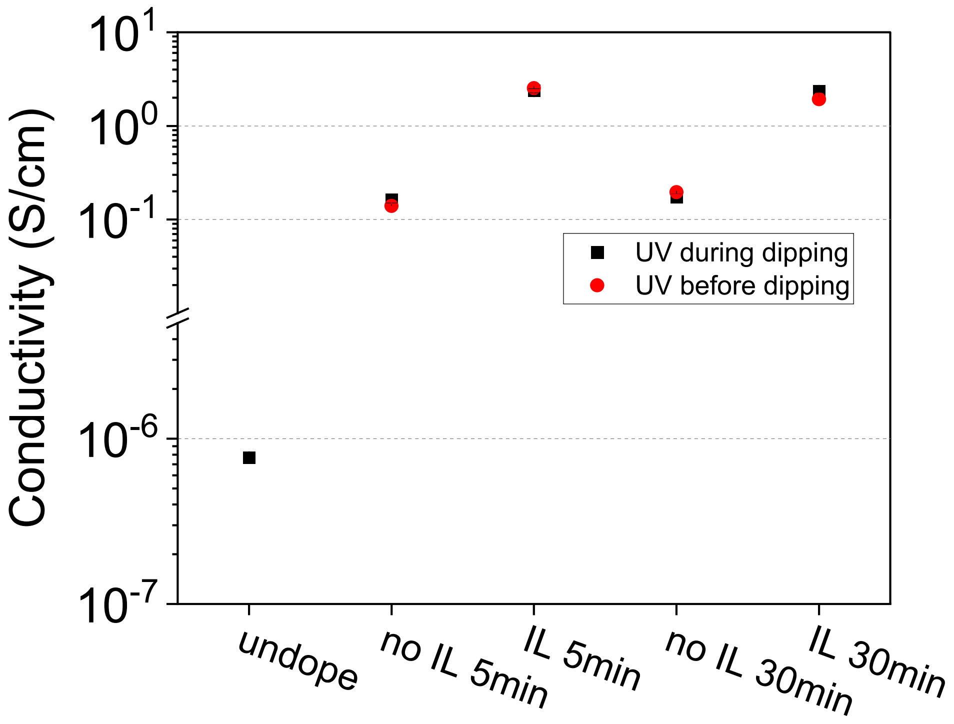

Fig. S6.

Dip doping results for BBL with UV during dipping and UV before dipping. In all cases, initial concentration is Mes-Acr^+: 1 mg/mL, DIPEA: 1 µL/mL. UV-before dipping started with 1 hour illumination of the dopant solution before dipping.

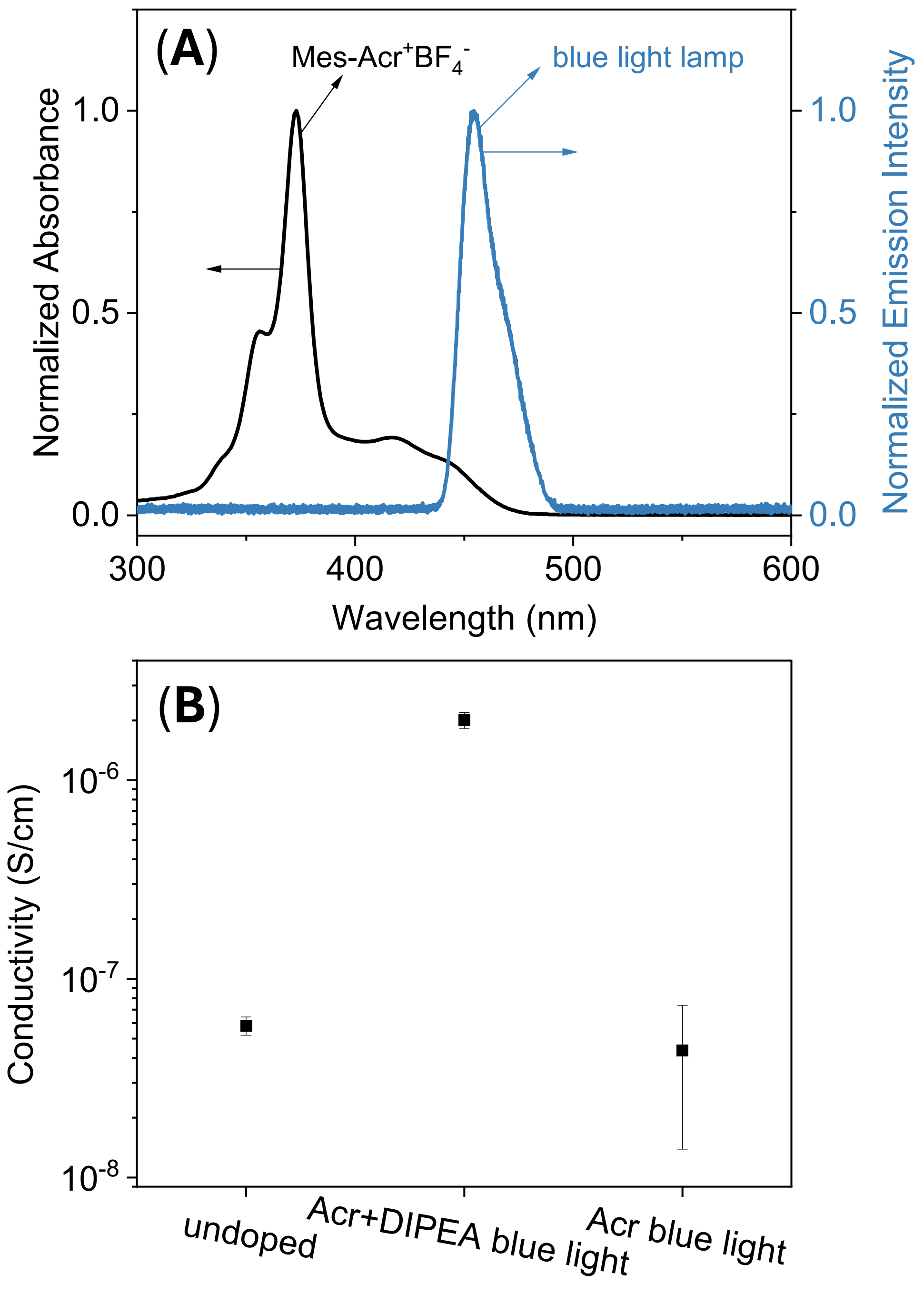

Fig. S7.

Dip doping under blue light illumination. (**A**) Normalized absorption spectrum of Mes-Acr$^+BF_4^-$ and emission spectrum of blue light source (34W Kessil H150-blue). (**B**) Dip doping for N2200 under blue light illumination for 30 mins. (Mes-Acr$^+BF_4^-$ 1 mg/mL, DIPEA 1 μL/mL)

Supplemental Note 1:

The conductivity achieved under the blue light illumination (**Fig. S**6B) is over an order of magnitude lower than that obtained under ultraviolet light (**Fig. 1C**). This difference in conductivity suggests different numbers of generated radicals, which is influenced by factors such as the number of incident photons and the absorption coefficient of Mes-$Acr^{+}BF4^{-}$ at the lamp's emission wavelength. The UV lamp (Ray B-100A high-intensity UV lamp, 100 W, 365 nm) primarily emits at 365 nm, whereas the blue light lamp (34W Kessil H150-blue) predominantly emits at 450 nm (**Fig. S6A**). Since the main absorption peak of Mes-$Acr^{+}BF_4^{-}$ occurs at 370 nm (largely overlapping with the emission of the UV-lamp), with significantly weaker absorption at 450 nm (where the blue lamp emits), the quantity of photo-generated Mes-Acr• radical under the blue light illumination should be substantially lower than under UV light with the same exposure time. According to the proposed mechanism of photoredox doping, the quantity of generated Mes-Acr• radical would decide the doping level and observed conductivity; thus, the observed conductivity would be lower when blue light is used.

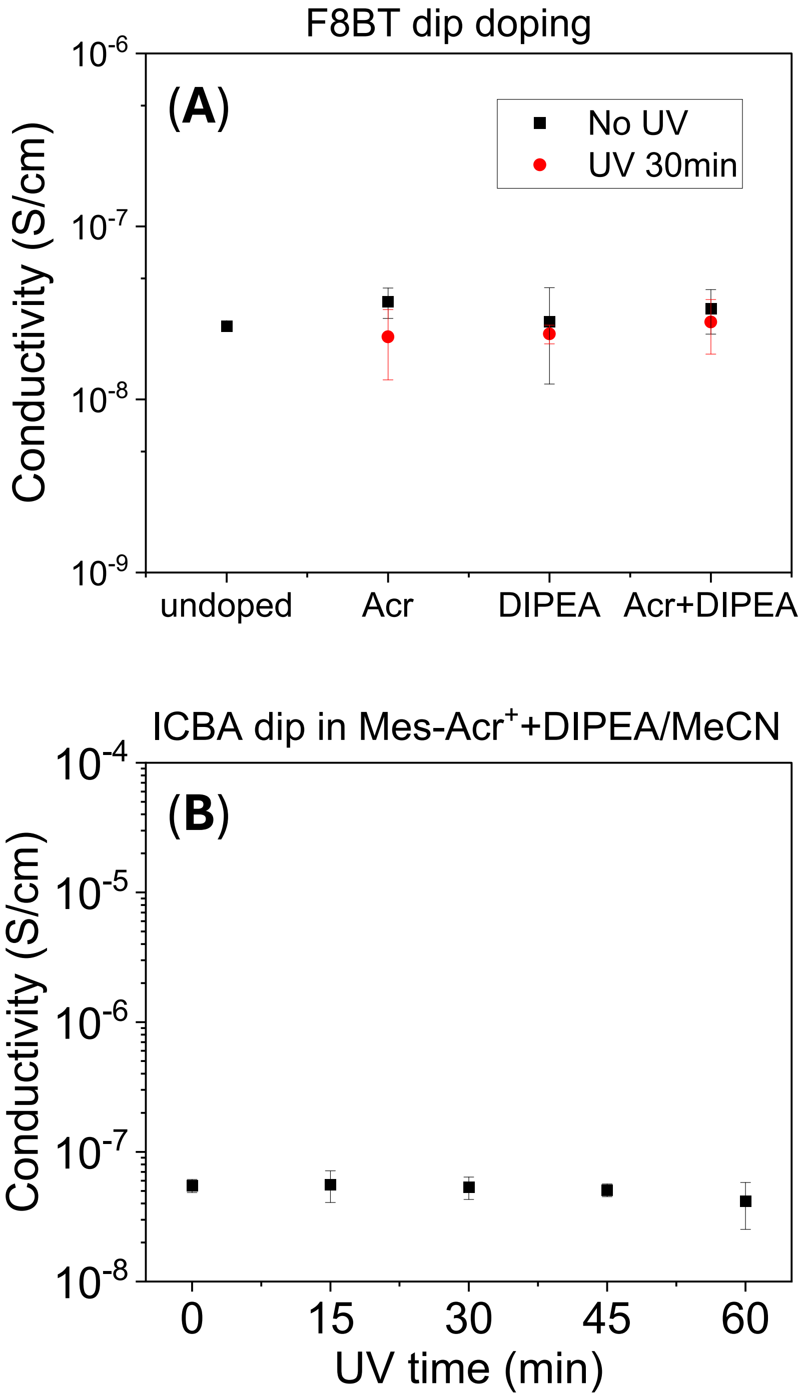

Fig. S8.

Dip doping result for F8BT and ICBA. (**A**) dip doping result for F8BT, (**B**) dip doping result for ICBA. In both cases, Mes-Acr$^+BF_4^-$ 1mg/mL, DIPEA 1 μL/mL, with UV during dipping method.

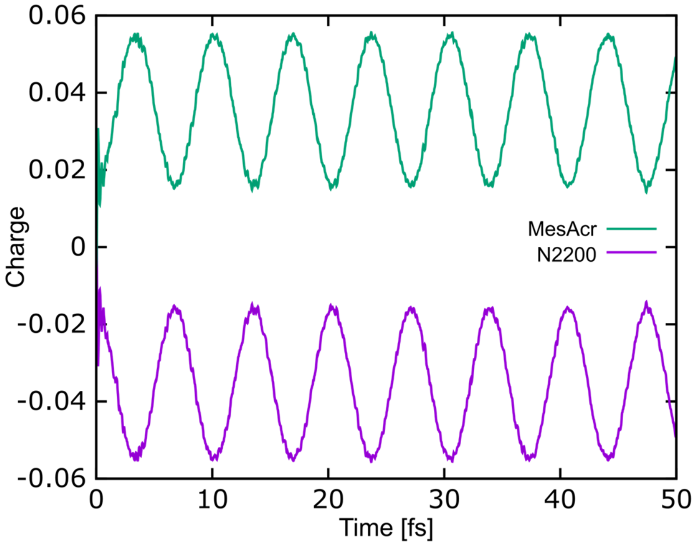

Fig. S9.

Time-dependent Mulliken charge localized in the Mes-Acr• and N2200 fragments starting from the neutral non-bonded [Mes-Acr•···N2200] complex.

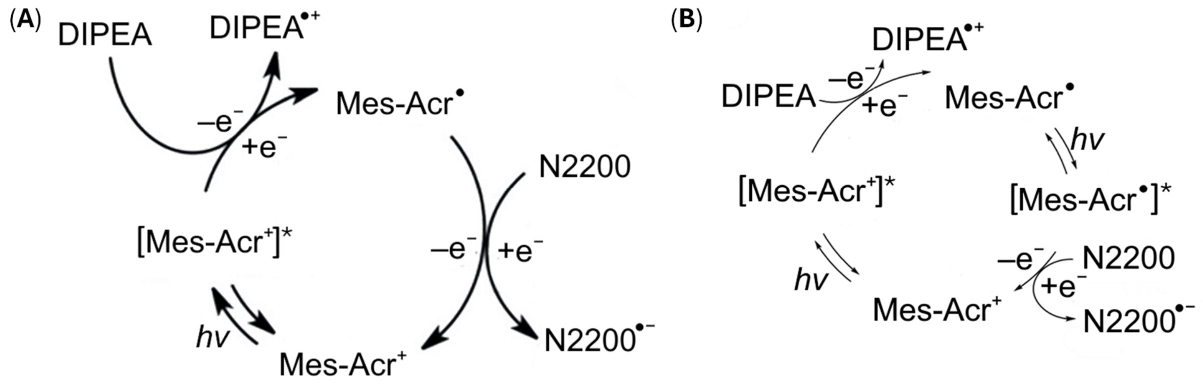

Fig. S10.

Proposed doping mechanism. (**A**) one-photon-one-electron process, (**B**) two-photon-two-electron process.

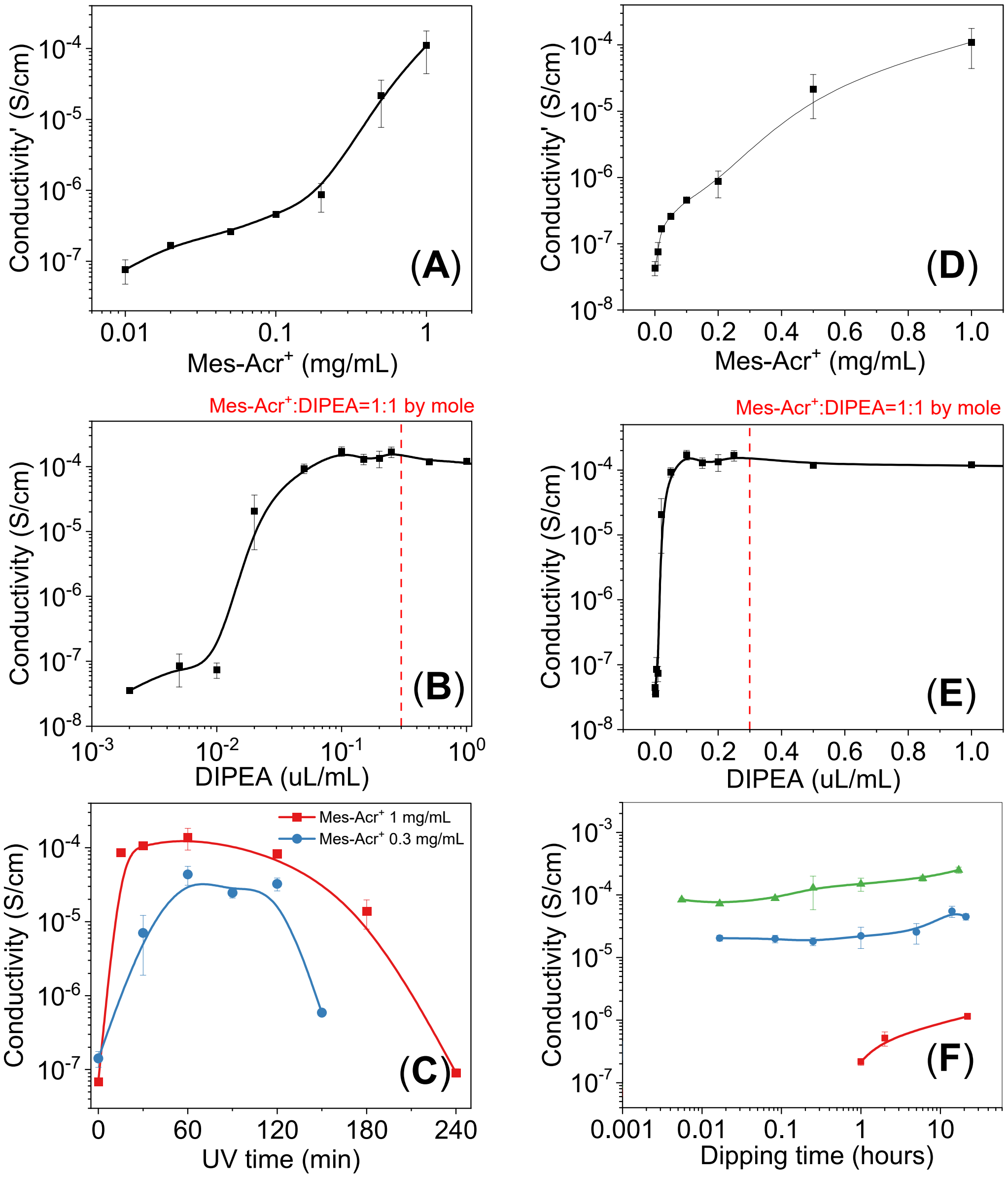

Fig. S11.

Concentration and dipping time dependence for N2200 with photoredox catalyst doping. The conductivity of doped N2200 film depends on the starting concentration of Mes-Acr$^+$ when DIPEA is in excess (UV during dipping), (**A**) log-log plot, (**D**) semi-log plot. When DIPEA is the limiting reagent, the conductivity of doped N2200 film depends on the concentration of DIPEA (UV during dipping). (**B**) log-log plot, (**E**) semi-log plot. Please note that molar equivalence of these two reagents at about 0.3 µL/mL DIPEA vs. 1 mg/mL Mes-Acr$^+$BF$_4^-$. Time-dependence of conductivity of N2200 film doped with different Mes-Acr$^+$ concentration, (**C**) log-log plot under 'UV during dipping' condition, (**F**) semi-log plot under 'UV before dipping' condition (i.e., 1 hour illumination of the dopant solution before dipping).

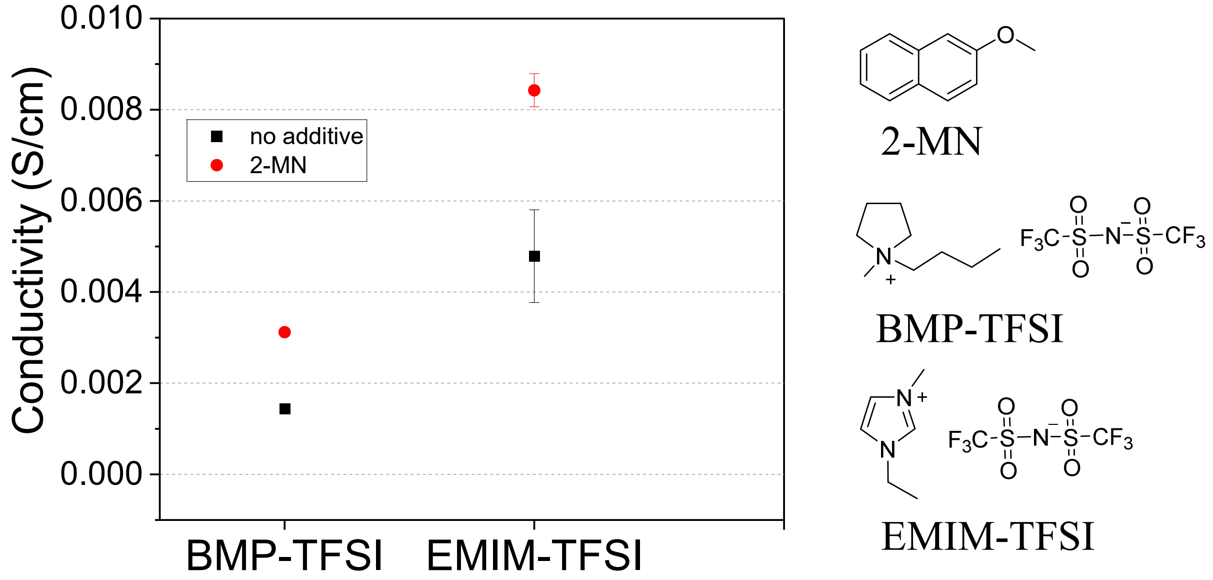

Fig. S12.

Conductivity of photocatalyst doped N2200 through UV before dipping method with additive 2-MN and different ionic liquid. 2-MN was added 150 wt% to N2200 and all films were thermal annealed at 80°C for 5 min before dip doping. Mes-Acr$^+$BF$_4^-$ is 1 mg/mL, DIPEA is 1 μL/mL, the ionic liquids were maintained 100:1 to Mes-Acr$^+$BF$_4^-$. The solution was under UV exposure for 2 hours then the UV light was switched off and dip the annealed polymer film in the solution for 30 min.

Supplemental Note 2:

It should be noted that conductivity is not only related to the carrier concentration but also the mobility of charge carriers in the film. The former is related to doping efficiency, while the latter is related to film morphology. Therefore, the processing conditions would largely affect the conductivity. Here, we are not focused on the optimization of processing conditions but the mechanism of the doping process. But we just show an example of the potential optimization would increase the conductivity. It was found that different ionic liquids would lead to different levels of final conductivity in n-type doping of N2200(*45*) and the additive (2-methoxynaphthalene, 2-MN) would largely improve the film morphology in organic solar cells.(*46*) We added 2-MN into the N2200 film and performed a little thermal annealing and we used 1-Ethyl-3-methylimidazolium bis(trifluoromethylsulfonyl)imide (EMIM-TFSI) instead of the BMP-TFSI we used previously. After that with UV before dipping method, we could achieve better conductivity of N2200 than we previously presented, as shown in Figure S12. Therefore, the processing conditions could change the conductivity a lot. Our diffusion model ignores the processing caused morphology change.

Diffusion in dip doping:

When the UV light is on during the dipping process (UV during dipping), four steps can happen as below:

Excitation: $\text{Mes-Acr}^{+}\text{+hv} \xrightarrow{k_{ex}} \text{Mes-Acr}^{+}*$ (1)

Emission: $\text{Mes-Acr}^{+}* \xrightarrow{k_{em}} \text{Mes-Acr}^{+}\text{+hv'}$ (2)

Eletron transfer: $\text{Mes-Acr}^{+}\text{*+DIPEA} \xrightarrow{k_{tr}} \text{Mes-Acr}^{\bullet} \text{ +DIPEA}^{\bullet+}$ (3)

Doping: $\text{Mes-Acr}^{\bullet}\text{+N2200} \xrightarrow{k_{d}} \text{Mes-Acr}^{+} \text{ +N2200}^{\bullet-}$ (4)

It should be noted that the real dopant is the radical Mes-Acr•, generated from step (4). The radical concentration is determined by (a) $\text{Mes-Acr}^{+}\text{BF}_4^{-}$ concentration, (b) DIPEA concentration, (c) light intensity and (d) the illumination time. Therefore, we can change the $\text{Mes-Acr}^{+}\text{BF}_4^{-}$ and DIPEA concentration to control the Mes-Acr• concentration.

Step (4) is a solid-liquid reaction which is likely controlled by diffusion. In the simplified 'UV before doping' process, steps 1 through 3 already happened to generate the Mes-Acr•, which would accumulate to have certain concentration of the Mes-Acr• (since Mes-Acr• appears to be sufficiently stable in solution*(18)* that would then accomplish the doping conjugated polymers via step 4. In this scenario, there are two key steps: a) radical generation process before dipping (when UV was on), and b) diffusion-controlled dip doping process (when UV was off)

(a) radical generation process before dipping (with UV on)

In this process, only steps (1) – (3) were involved to generate the Mes-Acr•.

At the beginning of the illumination, the # of Mes-Acr• is very small. After illumination starts, the concentration of Mes-Acr• would monotonically increase with time. When the illumination time is long enough, the concentration of Mes-Acr• would then be limited by the initial concentration of $\text{Mes-Acr}^{+}\text{BF}_4^{-}$ (or the initial concentration of DIPEA, if DIPEA is less than $\text{Mes-Acr}^{+}\text{BF}_4^{-}$). Therefore, it is reasonable to assume the concentration of Mes-Acr• would be proportional to initial concentration of $\text{Mes-Acr}^{+}\text{BF}_4^{-}$ after long enough UV light illumination time.

(b) diffusion controlled dip doping process (with UV off)

If we assume the density of N2200 is 1 g/cm^3, the 100 nm of N2200 film on 1.5 cm by 1.5 cm glass substrate will be 2.25×10^{-5} g. There are 2.27×10^{-8} mol of repeating unit of N2200 in the film (mole mass of the repeating unit is 989.5 g/mol). The total amount of $\text{Mes-Acr}^{+}\text{BF}_4^{-}$ (mole mass 573.51 g/mol) in 1.5 mL of 1 mg/mL Acr solution is 2.62×10^{-6} mol. As the # of $\text{Mes-Acr}^{+}\text{BF}_4^{-}$ in the solution is much larger (~100 x) than the # of repeating unit in the thin film, we can consider the radical Mes-Acr• concentration (which would be proportional to the # of $\text{Mes-Acr}^{+}\text{BF}_4^{-}$ in the solution, see above) is ***constant*** throughout the doping process. Therefore, the whole diffusion process is a diffusion problem as a semi-infinite solid with constant surface concentration.

According to Fick's second law, the relationship between diffusion time t and the dopant concentration C is:

$$\frac{\partial C}{\partial t} = D\frac{\partial^2 C}{\partial x^2}$$

where D is the diffusion constant, and the diffusion boundary condition is:

t=0: x=0, $C=C_s$; x>0, $C_x=0$
t>0: x=0, $C=C_s$

Where C_x is the concentration of dopant at distance x, C_0 is the concentration of dopant in the film before diffusion (which should be zero), C_s is the dopant concentration in the solution (which is constant during the doping process; see above argument).

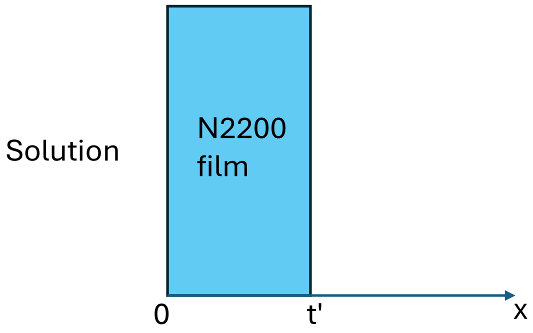

Fig. S13.
Instruction of the diffusion coordinate. t' is the thickness of the film, x is the distance from the liquid/solid interface into the N2200 film.

If we assume the diffusion did not reach the bottom of the film, it is a diffusion problem as a semi-infinite solid with constant surface concentration(*47*):

$$\frac{C_x - C_0}{C_s - C_0} = 1 - \operatorname{erf}\left(\frac{x}{2\sqrt{Dt}}\right)$$

Where erf is the Gaussian error function, $\operatorname{erf} z = \frac{2}{\sqrt{\pi}}\int_0^z e^{-t^2}\,dt.$

In our case, C_0=0 (there is no Mes-Acr$^{\bullet}$ in polymer at the beginning).

$$\frac{C_x}{C_s} = 1 - \operatorname{erf}\left(\frac{x}{2\sqrt{Dt}}\right)$$

At time t, the total number of dopants (e.g., Mes-Acr$^{\bullet}$) that would go across the area (A) of the film into the polymer film is:

$$N_d = A \times \int_0^{t'} C_x \, dx = A \times C_s \int_0^{t'} \left(1 - \mathrm{erf}\left(\frac{x}{2\sqrt{Dt}}\right)\right) dx$$

As the intrinsic carrier density in organic semiconductors is very low, we could use the dopant density ($n_d = N_d/At'$) as the carrier density n.

Assume the mobility μ and morphology of the film do not change, the average conductivity of the film is

$$\sigma = en\mu = \frac{eN_d\mu}{At'} = \frac{e\mu C_s}{t'} \times \int_0^{t'} \left(1 - \mathrm{erf}\left(\frac{x}{2\sqrt{Dt}}\right)\right) dx$$

If the thickness t' and the diffusion time t were kept constant across the experiments, then the integration will be a constant K.

Therefore, $\sigma = \frac{Ke\mu}{t'} \times C_s$, where $K = \int_0^{t'} \left(1 - \mathrm{erf}\left(\frac{x}{2\sqrt{Dt}}\right)\right) dx$

This last equation clearly shows that the conductivity is roughly proportional to the ***initial*** concentration of Mes-Acr$^{\bullet}$ (which would be proportional to the ***initial*** Mes-Acr^{+}BF_4^{-} concentration in the solution, see above argument). Thus, controlling the initial concentration of Mes-Acr^{+}BF_4^{-} can have a ***direct*** effect on the measured conductivity (e.g., a linear relationship if all assumptions above were correct), as shown in **Figure 4B.**

REFERENCES AND NOTES

1. A. D. Scaccabarozzi, A. Basu, F. Aniés, J. Liu, O. Zapata-Arteaga, R. Warren, Y. Firdaus, M. I. Nugraha, Y. Lin, M. Campoy-Quiles, N. Koch, C. Müller, L. Tsetseris, M. Heeney, T. D. Anthopoulos, Doping approaches for organic semiconductors. *Chem. Rev.* **122**, 4420–4492 (2022).

2. S. R. Marder, S. Barlow, Reaction mechanisms for electrical doping of organic semiconductors using complex dopants. *Chem. Phys. Rev.* **5**, 021303 (2024).

3. C. G. Tang, M. N. Syafiqah, Q.-M. Koh, C. Zhao, J. Zaini, Q.-J. Seah, M. J. Cass, M. J. Humphries, I. Grizzi, J. H. Burroughes, R.-Q. Png, L.-L. Chua, P. K. H. Ho, Multivalent anions as universal latent electron donors. *Nature* **573**, 519–525 (2019).

4. S.-G. Kim, G. C. Fish, E. Socie, A. T. Terpstra, D.-A. Park, K. Zhu, M. Grätzel, J.-E. Moser, N.-G. Park, Photo-doping of spiro-OMeTAD for highly stable and efficient perovskite solar cells. *Joule* **8**, 1707–1722 (2024).

5. T. Zhang, F. Wang, H.-B. Kim, I.-W. Choi, C. Wang, E. Cho, R. Konefal, Y. Puttisong, K. Terado, L. Kobera, M. Chen, M. Yang, S. Bai, B. Yang, J. Suo, S.-C. Yang, X. Liu, F. Fu, H. Yoshida, W. M. Chen, J. Brus, V. Coropceanu, A. Hagfeldt, J.-L. Brédas, M. Fahlman, D. S. Kim, Z. Hu, F. Gao, Ion-modulated radical doping of spiro-OMeTAD for more efficient and stable perovskite solar cells. *Science* **377**, 495–501 (2022).

6. N. Sakai, R. Warren, F. Zhang, S. Nayak, J. Liu, S. V. Kesava, Y.-H. Lin, H. S. Biswal, X. Lin, C. Grovenor, T. Malinauskas, A. Basu, T. D. Anthopoulos, V. Getautis, A. Kahn, M. Riede, P. K. Nayak, H. J. Snaith, Adduct-based p-doping of organic semiconductors. *Nat. Mater.* **20**, 1248–1254 (2021).

7. J. Liu, B. van der Zee, R. Alessandri, S. Sami, J. Dong, M. I. Nugraha, A. J. Barker, S. Rousseva, L. Qiu, X. Qiu, N. Klasen, R. C. Chiechi, D. Baran, M. Caironi, T. D. Anthopoulos, G. Portale, R. W. A. Havenith, S. J. Marrink, J. C. Hummelen, L. J. A. Koster, N-type organic thermoelectrics: Demonstration of ZT > 0.3. *Nat. Commun.* **11**, 5694 (2020).

8. B. Russ, A. Glaudell, J. J. Urban, M. L. Chabinyc, R. A. Segalman, Organic thermoelectric materials for energy harvesting and temperature control. *Nat. Rev. Mater.* **1**, 16050 (2016).

9. X. Yan, M. Xiong, X.-Y. Deng, K.-K. Liu, J.-T. Li, X.-Q. Wang, S. Zhang, N. Prine, Z. Zhang, W. Huang, Y. Wang, J.-Y. Wang, X. Gu, S. K. So, J. Zhu, T. Lei, Approaching disorder-tolerant semiconducting polymers. *Nat. Commun.* **12**, 5723 (2021).

10. X. Lin, B. Wegner, K. M. Lee, M. A. Fusella, F. Zhang, K. Moudgil, B. P. Rand, S. Barlow, S. R. Marder, N. Koch, A. Kahn, Beating the thermodynamic limit with photo-activation of n-doping in organic semiconductors. *Nat. Mater.* **16**, 1209–1215 (2017).

11. B. D. Naab, S. Guo, S. Olthof, E. G. B. Evans, P. Wei, G. L. Millhauser, A. Kahn, S. Barlow, S. R. Marder, Z. Bao, Mechanistic study on the solution-phase n-doping of 1,3-dimethyl-2-aryl-2,3-dihydro-1H-benzoimidazole derivatives. *J. Am. Chem. Soc.* **135**, 15018–15025 (2013).

12. X. Zhao, D. Madan, Y. Cheng, J. Zhou, H. Li, S. M. Thon, A. E. Bragg, M. E. DeCoster, P. E. Hopkins, H. E. Katz, High conductivity and electron-transfer validation in an n-type fluoride-anion-doped polymer for thermoelectrics in air. *Adv. Mater.* **29**, 1606928 (2017).

13. O. Bardagot, C. Aumaître, A. Monmagnon, J. Pécaut, P.-A. Bayle, R. Demadrille, Revisiting doping mechanisms of n-type organic materials with N-DMBI for thermoelectric applications: Photo-activation, thermal activation, and air stability. *Appl. Phys. Lett.* **118**, 203904 (2021).

14. A. Tlili, S. Lakhdar, Acridinium salts and cyanoarenes as powerful photocatalysts: Opportunities in organic synthesis. *Angew. Chem. Int. Ed.* **60**, 19526–19549 (2021).

15. M. Melchionna, P. Fornasiero, Updates on the roadmap for photocatalysis. *ACS Catal.* **10**, 5493–5501 (2020).

16. C. K. Prier, D. A. Rankic, D. W. C. MacMillan, Visible light photoredox catalysis with transition metal complexes: Applications in organic synthesis. *Chem. Rev.* **113**, 5322–5363 (2013).

17. N. A. Romero, D. A. Nicewicz, Organic photoredox catalysis. *Chem. Rev.* **116**, 10075–10166 (2016).

18. I. A. MacKenzie, L. Wang, N. P. R. Onuska, O. F. Williams, K. Begam, A. M. Moran, B. D. Dunietz, D. A. Nicewicz, Discovery and characterization of an acridine radical photoreductant. *Nature* **580**, 76–80 (2020).

19. W. Jin, C.-Y. Yang, R. Pau, Q. Wang, E. K. Tekelenburg, H.-Y. Wu, Z. Wu, S. Y. Jeong, F. Pitzalis, T. Liu, Q. He, Q. Li, J.-D. Huang, R. Kroon, M. Heeney, H. Y. Woo, A. Mura, A. Motta, A. Facchetti, M. Fahlman, M. A. Loi, S. Fabiano, Photocatalytic doping of organic semiconductors. *Nature* **630**, 96–101 (2024).

20. D. Yuan, W. Liu, X. Zhu, Efficient and air-stable n-type doping in organic semiconductors. *Chem. Soc. Rev.* **52**, 3842–3872 (2023).

21. S. Griggs, A. Marks, H. Bristow, I. McCulloch, n-Type organic semiconducting polymers: Stability limitations, design considerations and applications. *J. Mater. Chem. C* **9**, 8099–8128 (2021).

22. H. Guo, C.-Y. Yang, X. Zhang, A. Motta, K. Feng, Y. Xia, Y. Shi, Z. Wu, K. Yang, J. Chen, Q. Liao, Y. Tang, H. Sun, H. Y. Woo, S. Fabiano, A. Facchetti, X. Guo, Transition metal-catalysed molecular n-doping of organic semiconductors. *Nature* **599**, 67–73 (2021).

23. Y. Yamashita, J. Tsurumi, M. Ohno, R. Fujimoto, S. Kumagai, T. Kurosawa, T. Okamoto, J. Takeya, S. Watanabe, Efficient molecular doping of polymeric semiconductors driven by anion exchange. *Nature* **572**, 634–638 (2019).

24. R. A. Schlitz, F. G. Brunetti, A. M. Glaudell, P. L. Miller, M. A. Brady, C. J. Takacs, C. J. Hawker, M. L. Chabinyc, Solubility-limited extrinsic n-type doping of a high electron mobility polymer for thermoelectric applications. *Adv. Mater.* **26**, 2825–2830 (2014).

25. G. Ye, J. Liu, X. Qiu, S. Stäter, L. Qiu, Y. Liu, X. Yang, R. Hildner, L. J. A. Koster, R. C. Chiechi, Controlling n-type molecular doping via regiochemistry and polarity of pendant

groups on low band gap donor–acceptor copolymers. *Macromolecules* **54**, 3886–3896 (2021).

26. J. Liu, L. Qiu, R. Alessandri, X. Qiu, G. Portale, J. Dong, W. Talsma, G. Ye, A. A. Sengrian, P. C. T. Souza, M. A. Loi, R. C. Chiechi, S. J. Marrink, J. C. Hummelen, L. J. A. Koster, Enhancing molecular n-type doping of donor–acceptor copolymers by tailoring side chains. *Adv. Mater.* **30**, 1704630 (2018).

27. D. Rosas Villalva, S. Singh, L. A. Galuska, A. Sharma, J. Han, J. Liu, M. A. Haque, S. Jang, A. H. Emwas, L. J. A. Koster, X. Gu, B. C. Schroeder, D. Baran, Backbone-driven host–dopant miscibility modulates molecular doping in NDI conjugated polymers. *Mater. Horiz.* **9**, 500–508 (2022).

28. A. Babel, S. A. Jenekhe, High electron mobility in ladder polymer field-effect transistors. *J. Am. Chem. Soc.* **125**, 13656–13657 (2003).

29. J. Guo, L. Q. Flagg, D. K. Tran, S. E. Chen, R. Li, N. B. Kolhe, R. Giridharagopal, S. A. Jenekhe, L. J. Richter, D. S. Ginger, Hydration of a side-chain-free n-type semiconducting ladder polymer driven by electrochemical doping. *J. Am. Chem. Soc.* **145**, 1866–1876 (2023).

30. N. Holmberg-Douglas, D. A. Nicewicz, Photoredox-catalyzed C–H functionalization reactions. *Chem. Rev.* **122**, 1925–2016 (2022).

31. P. Hohenberg, W. Kohn, Inhomogeneous electron gas. *Phys. Rev.* **136**, B864–B871 (1964).

32. W. Kohn, L. J. Sham, Self-consistent equations including exchange and correlation effects. *Phys. Rev.* **140**, A1133–A1138 (1965).

33. E. Runge, E. K. U. Gross, Density-functional theory for time-dependent systems. *Phys. Rev. Lett.* **52**, 997–1000 (1984).

34. J. P. Perdew, K. Burke, M. Ernzerhof, Generalized gradient approximation made simple. *Phys. Rev. Lett.* **77**, 3865–3868 (1996).

35. C. Adamo, V. Barone, Toward reliable density functional methods without adjustable parameters: The PBE0 model. *J. Chem. Phys.* **110**, 6158–6170 (1999).

36. M. J. Frisch, G. W. Trucks, H. B. Schlegel, G. E. Scuseria, M. A. Robb, J. R. Cheeseman, G. Scalmani, V. Barone, G. A. Petersson, H. Nakatsuji, X. Li, M. Caricato, A. V. Marenich, J. Bloino, B. G. Janesko, R. Gomperts, B. Mennucci, H. P. Hratchian, J. V. Ortiz, A. F. Izmaylov, J. L. Sonnenberg, Williams, F. Ding, F. Lipparini, F. Egidi, J. Goings, B. Peng, A. Petrone, T. Henderson, D. Ranasinghe, V. G. Zakrzewski, J. Gao, N. Rega, G. Zheng, W. Liang, M. Hada, M. Ehara, K. Toyota, R. Fukuda, J. Hasegawa, M. Ishida, T. Nakajima, Y. Honda, O. Kitao, H. Nakai, T. Vreven, K. Throssell, J. A. Montgomery Jr., J. E. Peralta, F. Ogliaro, M. J. Bearpark, J. J. Heyd, E. N. Brothers, K. N. Kudin, V. N. Staroverov, T. A. Keith, R. Kobayashi, J. Normand, K. Raghavachari, A. P. Rendell, J. C. Burant, S. S. Iyengar, J. Tomasi, M. Cossi, J. M. Millam, M. Klene, C. Adamo, R. Cammi, J. W. Ochterski, R. L. Martin, K. Morokuma, O. Farkas, J. B. Foresman, D. J. Fox, Gaussian 16 Rev. J.14+. Wallingford, CT (2020).

37. S. Grimme, S. Ehrlich, L. Goerigk, Effect of the damping function in dispersion corrected density functional theory. *J. Comput. Chem.* **32**, 1456–1465 (2011).

38. F. Weigend, R. Ahlrichs, Balanced basis sets of split valence, triple zeta valence and quadruple zeta valence quality for H to Rn: Design and assessment of accuracy. *Phys. Chem. Chem. Phys.* **7**, 3297–3305 (2005).

39. F. Weigend, M. Häser, H. Patzelt, R. Ahlrichs, RI-MP2: Optimized auxiliary basis sets and demonstration of efficiency. *Chem. Phys. Lett.* **294**, 143–152 (1998).

40. A. V. Marenich, C. J. Cramer, D. G. Truhlar, Universal solvation model based on solute electron density and on a continuum model of the solvent defined by the bulk dielectric constant and atomic surface tensions. *J. Phys. Chem. B.* **113**, 6378–6396 (2009).

41. J. Tomasi, B. Mennucci, R. Cammi, Quantum mechanical continuum solvation models. *Chem. Rev.* **105**, 2999–3094 (2005).

42. W. Liang, C. T. Chapman, X. Li, Efficient first-principles electronic dynamics. *J. Chem. Phys.* **134**, 184102 (2011).

43. D. B. Williams-Young, A. Petrone, S. Sun, T. F. Stetina, P. Lestrange, C. E. Hoyer, D. R. Nascimento, L. Koulias, A. Wildman, J. Kasper, J. J. Goings, F. Ding, A. E. DePrince III, E. F. Valeev, X. Li, The Chronus Quantum software package. *WIREs Comput. Mol. Sci.* **10**, e1436 (2020).

44. P. D. Nguyen, F. Ding, S. A. Fischer, W. Liang, X. Li, Solvated first-principles excited-state charge-transfer dynamics with time-dependent polarizable continuum model and solvent dielectric relaxation. *J. Phys. Chem. Lett.* **3**, 2898–2904 (2012).

45. X. Zhao, M. Alsufyani, J. Tian, Y. Lin, S. Y. Jeong, H. Y. Woo, Y. Yin, I. McCulloch, High efficiency n-type doping of organic semiconductors by cation exchange. *Adv. Mater.* **36**, e2412811 (2024).

46. J. Song, Y. Li, Y. Cai, R. Zhang, S. Wang, J. Xin, L. Han, D. Wei, W. Ma, F. Gao, Y. Sun, Solid additive engineering enables high-efficiency and eco-friendly all-polymer solar cells. *Matter* **5**, 4047–4059 (2022).

47. "Diffusion in dilute solutions," in *Diffusion: Mass Transfer in Fluid Systems*, Cussler, E. L. Ed., Cambridge Series in Chemical Engineering (Cambridge Univ. Press, ed. 3, 2009), pp. 13–55.
